# Tunable Properties of Nature-Inspired *N*,*N*′-Alkylated Riboflavin Semiconductors

**DOI:** 10.3390/molecules26010027

**Published:** 2020-12-23

**Authors:** Jan Richtar, Lucia Ivanova, Dong Ryeol Whang, Cigdem Yumusak, Dominik Wielend, Martin Weiter, Markus Clark Scharber, Alexander Kovalenko, Niyazi Serdar Sariciftci, Jozef Krajcovic

**Affiliations:** 1 Materials Research Centre, Faculty of Chemistry, Brno University of Technology, Purkyňova 118, 612 00 Brno, Czech Republic; xcrichtar@fch.vut.cz (J.R.); xcivanova@vutbr.cz (L.I.); weiter@fch.vut.cz (M.W.); kovalenko.alx@gmail.com (A.K.); 2 Linz Institute for Organic Solar Cells (LIOS), Physical Chemistry, Johannes Kepler University Linz, Altenbergerstraße 69, 4040 Linz, Austria; whang@hnu.kr (D.R.W.); cigdem.yumusak@jku.at (C.Y.); dominik.wielend@jku.at (D.W.); Markus_Clark.Scharber@jku.at (M.C.S.); Serdar.Sariciftci@jku.at (N.S.S.)

**Keywords:** soluble alkylated flavins, bio-inspired materials, organic electronics, side-chain engineering, fused-ring systems, riboflavin, materials Science, general

## Abstract

A series of novel soluble nature-inspired flavin derivatives substituted with short butyl and bulky ethyl-adamantyl alkyl groups was prepared via simple and straightforward synthetic approach with moderate to good yields. The comprehensive characterization of the materials, to assess their application potential, has demonstrated that the modification of the conjugated flavin core enables delicate tuning of the absorption and emission properties, optical bandgap, frontier molecular orbital energies, melting points, and thermal stability. Moreover, the thin films prepared thereof exhibit smooth and homogeneous morphology with generally high stability over time.

## 1. Introduction

Natural and nature-inspired π-conjugated organic materials progressively enrich the pool of state-of-the-art materials for organic electronics and bioelectronics [1,2,3,4,5,6,7,8,9]. Considering their biocompatibility, non-toxicity, versatility, and sustainability together with potential for low-cost production, it is apparent that they are promising materials for bio-organic semiconductor science and technology such as ultra-thin electronic platforms for surgical and point-of-care devices or medical diagnosis [10,11,12,13], soft robotics [14,15], biodegradable electronics [16,17,18], self-healing wearable electronics [19], or photocatalytic processes like water splitting within the artificial photosynthesis [20].

Flavins are a class of naturally occurring π-conjugated organic materials with remarkable structural and chemical versatility [21]. These molecules are ubiquitous cofactors for a variety of enzymatic redox reactions. They are capable of both one- and two-electron transfer processes and play an essential mediative role in coupling the two-electron oxidation of most organic substances to the one-electron transfers of the respiratory chain [22]. Consequently, due to their photocatalytic abilities, flavins are involved in numerous biological phenomena such as photosynthesis [23], light-dependent repair of DNA damage [24], or detoxification of the soils of aromatic pollutants [25]. Flavin adenine dinucleotide (FAD) is the abundant cofactor constituting integral part of numerous flavoenzymes. An illustrative example is ferredoxin-NADP reductase, which contains noncovalently bound FAD as a prosthetic group. Ferredoxin-NADP reductase belongs to oxidoreductase group of enzymes catalyzing the final step of photosynthesis [26]. Herein, FAD is responsible for two single-electron-transfer steps within the light-driven electron transport chain providing the NADPH necessary for CO_2_ assimilation in plants and cyanobacteria. From a chemical point of view, FAD and other flavins can exist in three different redox states: Oxidized, one-electron reduced radical (semiquinone), and fully reduced hydroquinone, that contain 18, 19, and 20 π-electrons, respectively, in a π orbital system constructed from 16*p* atomic orbitals [22]. The redox-active part of the FAD cofactor is the diazabutadiene moiety of the isoalloxazine ring of the riboflavin. Likewise, the biological role of flavin derivatives as efficient redox centers, their artificial application as cathode materials for rechargeable lithium and sodium batteries has been put forward [27,28,29]. Moreover, the ability to act as an effective redox center during charge transduction is preserved even at the structurally simpler pteridine-based molecules—lumichrome, alloxazine, and lumazine—providing battery systems with one of the highest gravimetric energy densities and cycling performance among organic-based molecules [27]. Furthermore, the flavin derivatives have been successfully applied in photovoltaics [30], molecular logic gates or molecular switching [31,32], non-linear optics [33], or visible-light photocatalysis [34].

The electrochemical and photophysical properties of flavins are influenced by the local environment [35], π–π stacking [36], coordination to metal ions [37,38], or hydrogen-bonding interactions [39,40]. On the contrary, the effect of the expanding of the π-conjugated system on the optical and electronic properties of flavins is less explored, although changes in electrochemical properties and bathochromic shifts of the absorption maxima are anticipated [41]. A study investigating a series of flavins with progressively expanded fused π-systems and a series of non-fused derivatives was reported [42]. A strong dependence of the frontier molecular orbitals (FMO) energies determined by cyclic voltammetry on the size of the fused π-conjugated system was observed. The LUMO energies increased with enlarging of the π-system, while they remained virtually invariable for the non-fused flavin derivatives with alloxazine π-conjugated core [42]. It was shown that expansion of the π-conjugated system provided tunable light absorption reaching up to 700 nm. A similar trend was also observed for progressively expanded *N*10-butyl substituted flavins [43]. Moreover, the studied materials provided high thermal stability reaching up to 443 °C [42]. The synthesized flavins were proposed as possible promising materials for biomimetic energy storage or photocatalytic oxygen evolution due to their tunable optical and electronic properties. On the other hand, the NH-unsubstituted flavin derivatives suffer from poor solubility in common organic solvents due to strong intermolecular hydrogen-bonding which limits their technological processability [42,44].

In the current study, we focused on overcoming the issue of the poor solubility and thus limited processability by introducing alkyl substituents at the nitrogen atoms in positions *N*1 and *N*3 of the alloxazinic core, which would block the intermolecular hydrogen bonding. Furthermore, the quality of the alkyl substituent can significantly alter other physico-chemical properties like thermal stability and molecular organization in the solid state [45]. Thus, we have prepared the series of 8 flavin derivatives with progressively expanded π-conjugated systems substituted with either a short linear butyl group or a bulky ethyl-adamantyl group in analogy to our previous work [46]. For instance, introduction of bulky ethyl-adamantyl substituents to para-bis(2-thienyl)phenylene trimers induced the formation of highly organized crystal structures due to hydrogen bonding between thienyl and adamantyl and molecular pairing of ethyl-adamantyl side groups [46]. Hence, a significant increase of the melting point of the material and its fluorescent quantum yield was observed compared to the methyl-substituted trimer. Herein, we present the synthetic approach towards the novel symmetrically *N*,*N*′-dialkylated flavins and their comprehensive physico-chemical and quantum-chemical characterization. The role of the modification of the initial alloxazine core by the expansion of the π-conjugated system and the effect of alkyl substitution was evaluated. Moreover, vacuum-processed thin films were characterized by optical, electrical, electrochemical, and morphological measurements. The presented study enriches the flavin family by *N*,*N*′-alkylated derivatives and thus possible applicability in organic electronics and bioelectronics.

## 2. Results and Discussion

### 2.1. Synthesis

**Scheme 1:**
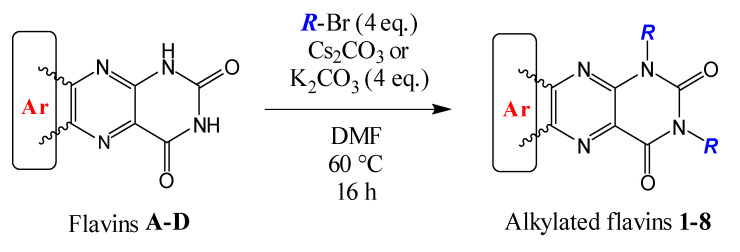
Synthesis of alkylated flavins **1**–**8**.

**Figure 1:**
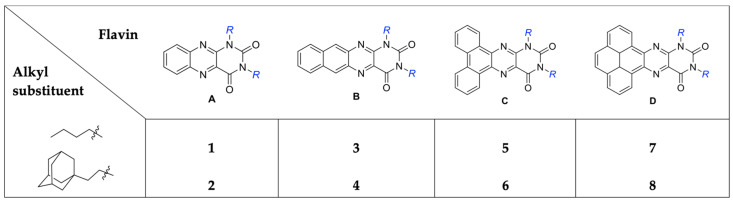
The structures of the compounds under investigation (flavins **1**–**8**). For the unsubstituted flavins, R = H.

The synthesis of symmetrically *N*,*N*′-dialkylated flavins was achieved via direct alkylation of the corresponding unsubstituted flavin derivatives (**A**–**D**, see Scheme 1 and Figure 1). The alkylation was realized by nucleophilic substitution at the activated amidic nitrogen atoms of the uracil moiety using cesium or potassium carbonates as bases and corresponding alkyl bromide as the alkylating agent. Given the increased solubility compared to NH-unalkylated derivatives, the alkylated flavins could be conveniently purified by silica gel column chromatography often involving multi-step purification. In cases of larger π-expanded systems, digestion in boiling lower alcohols followed. The syntheses provided the target compounds **1**–**8** (Figure 1) in moderate to good yields while the reaction conditions were not extensively optimized. The structure and purity of the target flavin derivatives was analyzed by ^1^H-NMR and elementary analysis.

### 2.2. Thermal Properties

TGA measurements have proved the high thermal stability of the flavin derivatives reaching 475 °C for compounds **6** and **8** (see Table 1). The thermal stability depends on the size of the π-conjugated system and on the nature of the alkyl substitution. The thermal stability of alkylated flavins generally increases with expanding the π-conjugated system following the same trend as for the unsubstituted flavins **A**–**D** (Figure 1). Comparing the effect of the alkyl substitution, the thermal stability of the butyl-substituted flavins (compounds **1**, **3**, **5**, and **7**) is lower compared to the ethyl-adamantyl-substituted counterparts (compounds **2**, **4**, **6**, and **8**). We assume that this observation can be reasoned by the effect of bulky rigid ethyl-adamantyl groups that can contribute to thermal stability by stronger intermolecular Van der Waals interactions in the solid state compared to short linear butyl groups at both the higher decomposition temperatures and higher melting points [46,47]. The contribution of ethyl-adamantyl substitution is even more remarkable when comparing with the decomposition temperatures of the unsubstituted flavins with prevalent strong intermolecular H-bonding. The decomposition temperatures are comparable or even higher for alkylated flavins than the unsubstituted derivatives (e.g., 398 °C and 475 °C for flavins **C** and **6**, respectively). Similarly, the melting points for ethyl-adamantyl-substituted derivatives (compounds **2**, **4**, **6** and **8**) are significantly higher than those measured for butyl-substituted flavins (compounds **1**, **3**, **5** and **7**). It should be stated that most of the derivatives are stable up to temperatures exceeding 300 °C and compounds **4**, **6,** and **8** even higher than 400 °C. Such thermal stability that is not common for such alkyl-substituted organic small molecules, can greatly contribute to the applications that require high temperature processing.

### 2.3. Optical and Electrical Properties

The family of flavin molecules described in the literature is a group of pigments with colors ranging from yellow to orange [48]. The derivatives studied in this work are pale yellow (**1**, **2**, **5**, **6**), yellow (**4**, **7**, **8**), and orange (**3**) powders.

**Figure 2:**
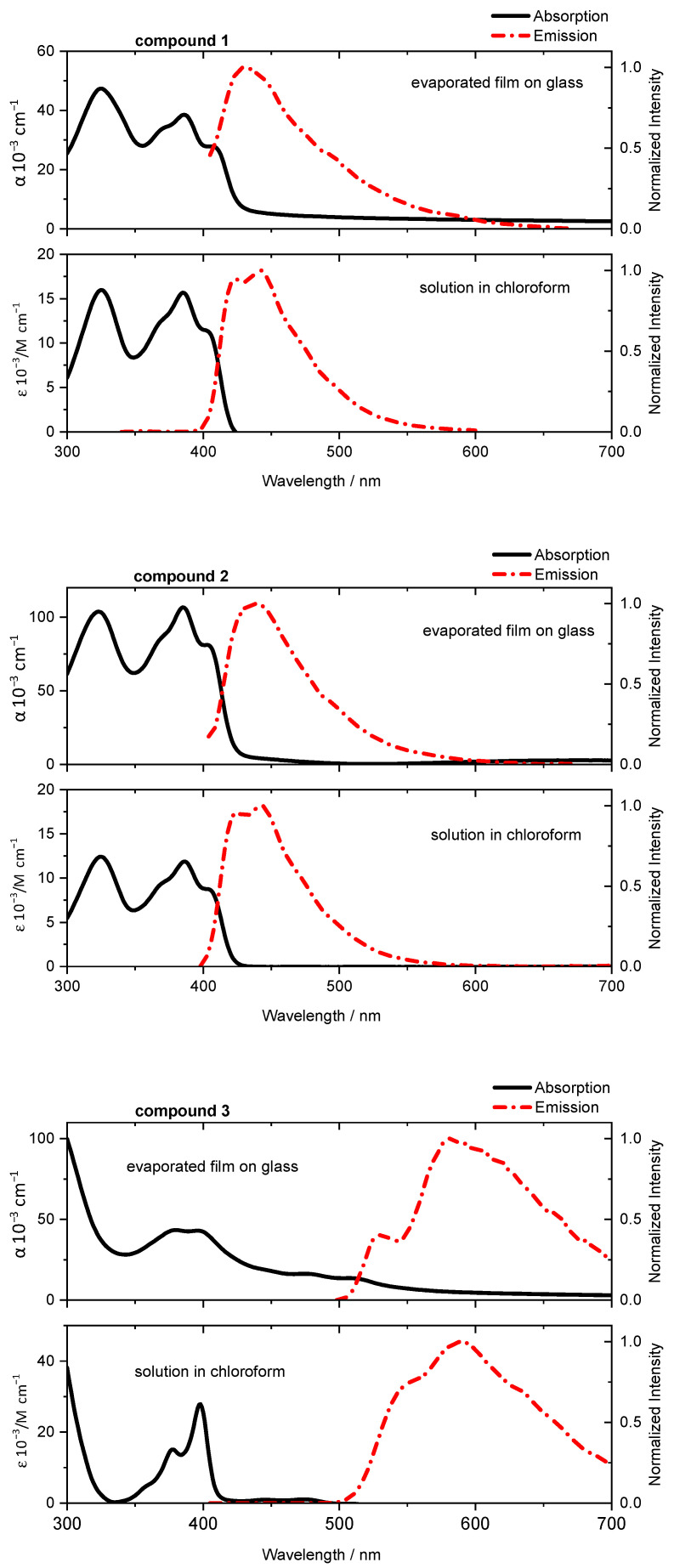

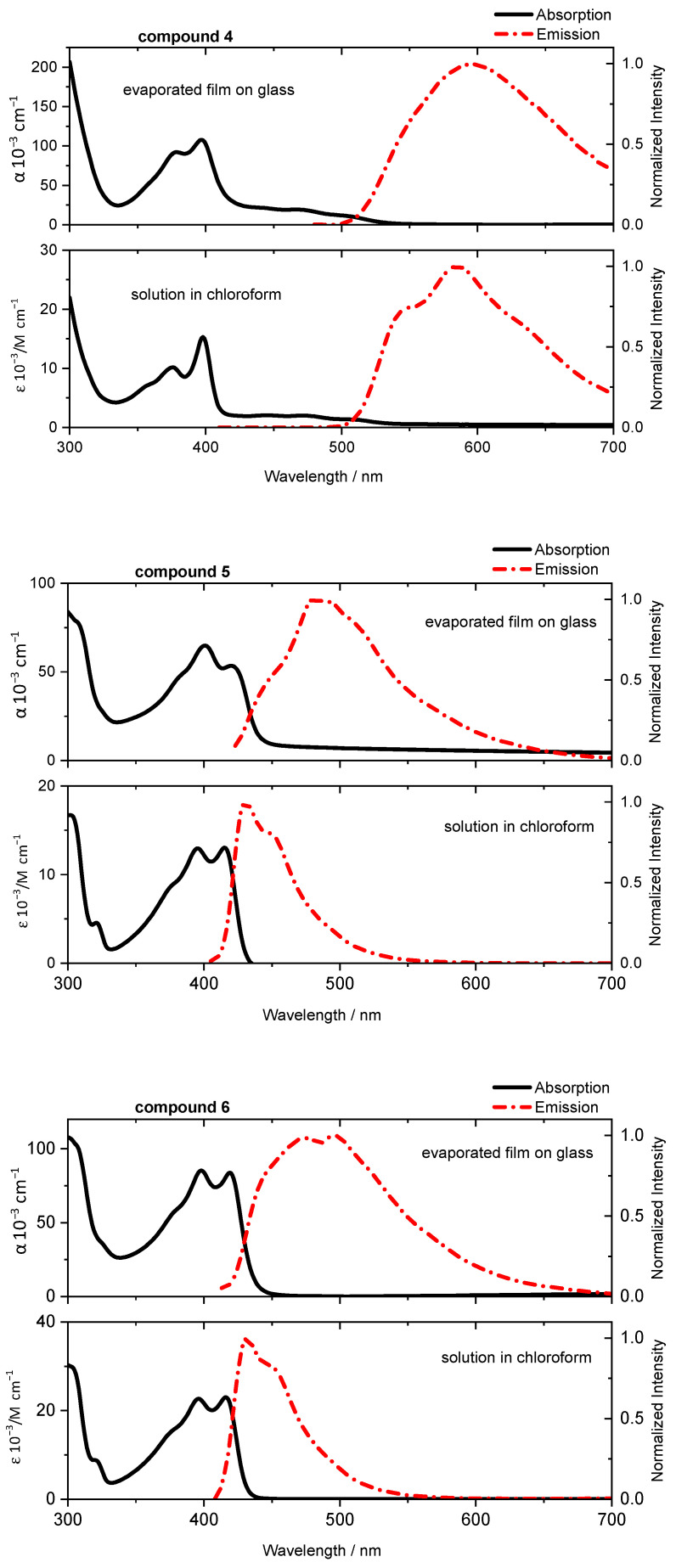

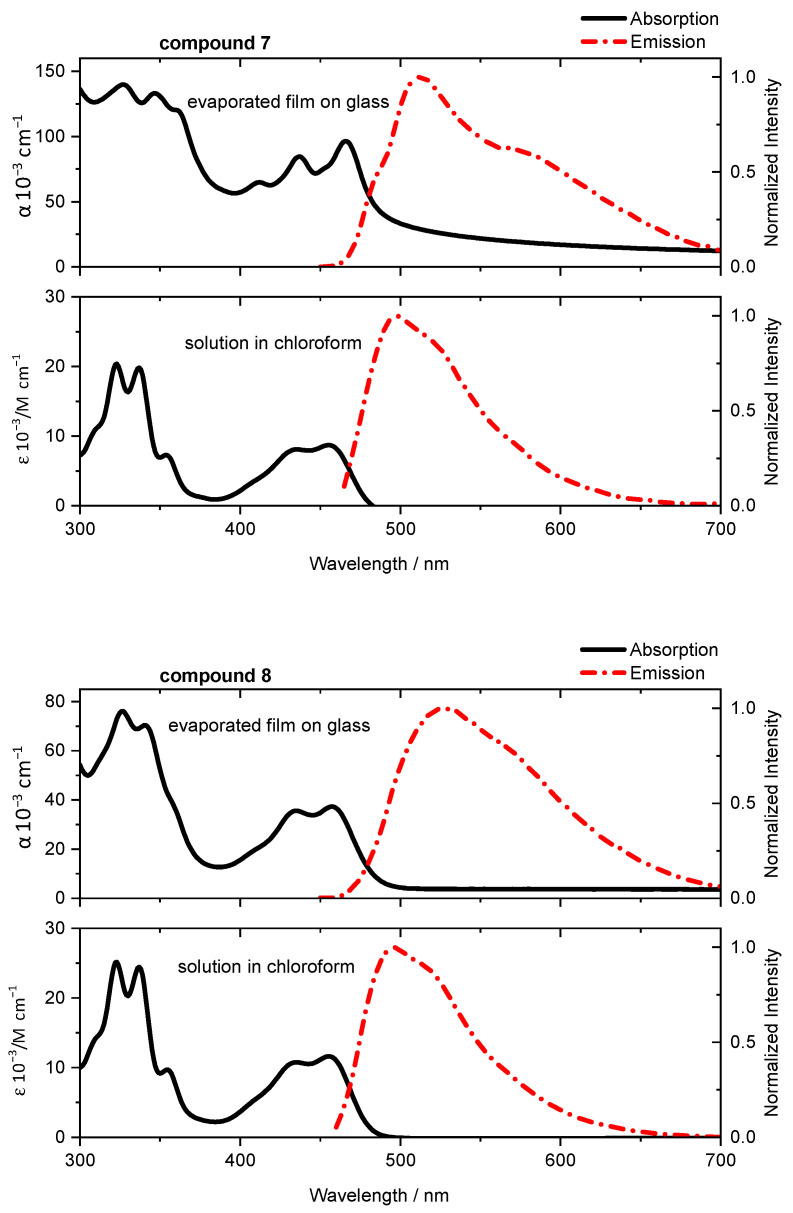
UV−Vis absorption and photoluminescence spectra of the compounds **1**–**8**.

The UV-Vis spectra (Figure 2) were collected to establish a relationship between the structure of the molecules and photophysical properties. Thus, the optical absorbance and photoluminescence of vacuum deposited thin films and diluted solutions of flavins in chloroform were measured.

The absorption spectra of both thin films and solutions of the flavin derivatives **1**–**8** show generally vibronic structure except compound **3** in solid-state, that is less resolved in terms of vibronic features. On the other hand, the vibronic structure is often less pronounced in the photoluminescence spectra. The gradual bathochromic shift in the absorption maxima is observed with increase in the size of the conjugated system. From the optical measurement results, it appears that variation of the structure of the flavins does not change the aggregation tendency of the molecules significantly.

**Figure 3:**
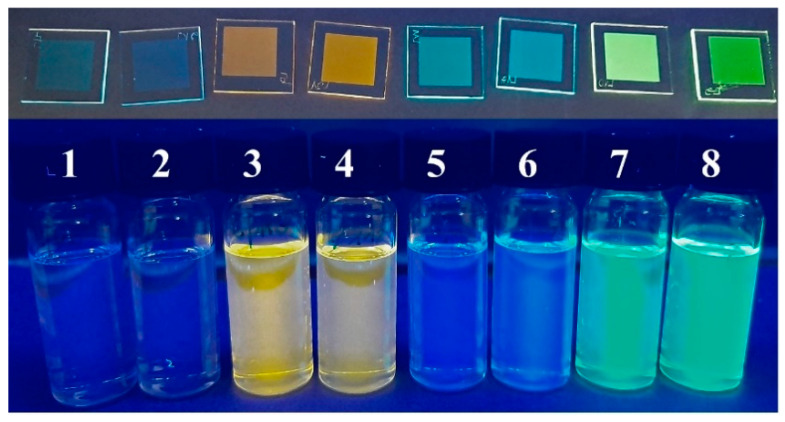
Thin films and solutions of the alkylated flavin derivatives illuminated with monochromatic light at 366 nm.

The pairs of analogous molecules with the same conjugated system and different side chains in solid-state (resp. solutions) produce the similar shape of the spectra when measuring both absorbance and emission varying in the position of local maxima (Table 2) and values of absorptivity coefficient (resp. molar absorption coefficient). Self-evidently analogues emit concordant color of the light in both forms as it is demonstrated in Figure 3.

When comparing the corresponding spectra of each molecule in both solid-state and solution, it is visible to proclaim that compounds **1** and **2**—the molecules with conjugated system extended by benzene moiety—behave in the same way while absorbing the photon in both forms. The photoluminescence peaks of solution spectra have vibronic features more distinguished.

Flavin derivatives with naphthalene moiety (compounds **3** and **4**) exhibit equal values of absorption maxima in both solid state and solution (Table 2). The emission maxima in the solution are shifted 10 nm bathochromically and 13 nm hypsochromically compared to the thin films for **3** and **4**, respectively. The flavin derivatives **4**–**6** show generally a larger hypsochromic shift of the emission maxima in the solid state compared to the solution, the most significant one being present in the emission spectra of flavins **5** and **6** in the chloroform.

In the case of the photoluminescence quantum yields, all the molecules show low values reaching maximum 2% (Table 2).

To investigate the electrical charge transport properties of the flavin derivatives, organic field effect transistors (OFET) were fabricated in a bottom-gate, bottom-contact geometry on highly *n*-doped silicon substrates, consisting of a 90 nm thermally grown silicon oxide (SiO_2_) insulating layer with pre-patterned interdigitated gold source and drain electrodes on a 10 nm ITO adhesion layer. The 80 nm of flavin derivatives were vacuum evaporated by physical vapor deposition (PVD) technique on top of the pre-treated substrates. The flavin OFETs showed *p*-type behavior under negative gate bias. The minor hole field-effect mobilities were observed for the phenanthrene and pyrene-derived flavins **5**–**8** with a maximum hole mobility of 6.5 × 10^−8^ cm^2^ V^−1^ s^−1^ for the flavin derivative **8** (further values see Table S3 in SI).

### 2.4. DFT Modeling

**Figure 4:**
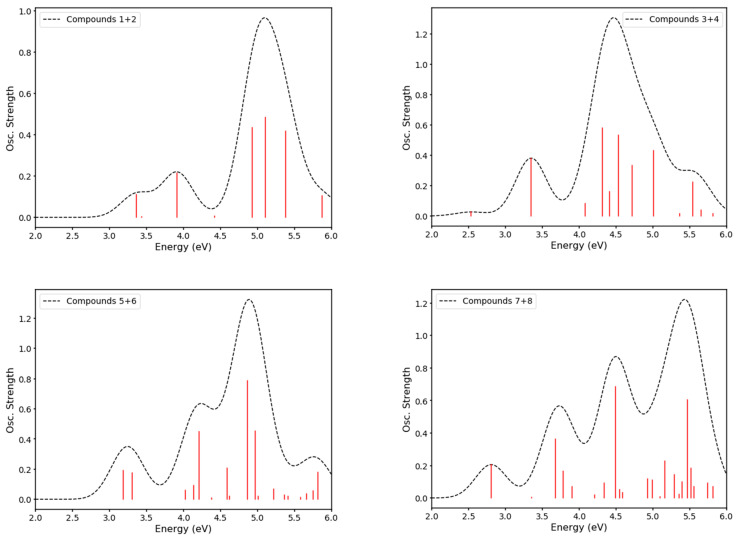
Vertical transitions calculated with TD-DFT and oscillator strength. Flavins denoted as **1+2** (phenyl moiety), **3+4** (naphthalene moiety), **5+6** (phenanthrene moiety), and **7+8** (pyrene moiety).

**Figure 5:**
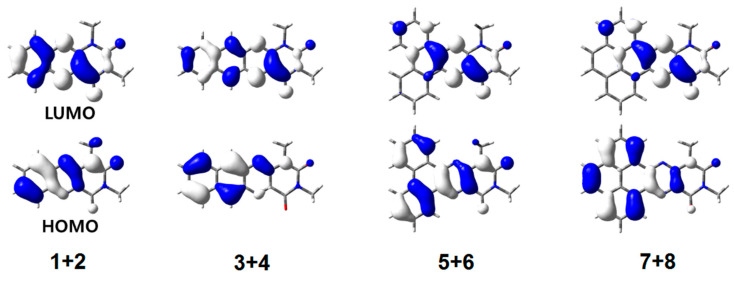
Highest occupied molecular orbital (HOMO) and lowest unoccupied molecular orbital (LUMO) contour diagrams for the alloxazine derivatives calculated with DFT. Alkyl chains considered only as α-CH_2_. Flavins denoted as **1+2** (phenyl moiety), **3+4** (naphthalene moiety), **5+6** (phenanthrene moiety), and **7+8** (pyrene moiety). An isodensity value of 0.03 electrons Å^−3^ was applied for the representations.

The optical properties of the alloxazine derivatives can be rationalized by quantum-chemical approach using density functional theory (DFT) methods. Vertical transitions calculated with TD-DFT are depicted in Figure 4 and the oscillator strengths well reproduces the experimental absorption results. The calculated characteristics of S_0_→S_1_ transitions are summarized in Table S2; all of them correspond to transitions from highest occupied molecular orbital (HOMO) to lowest unoccupied molecular orbital (LUMO). Figure 5 displays optimized geometries and molecular orbital (MO) contour diagrams for the molecules. All the LUMOs are localized on the pyrazine and dihydrouracil moiety. The HOMOs of **1**, **3**, and **4** are delocalized over the molecules. On the other hand, the HOMO of **2** has minimized contribution of pyrazine and dihydrouracil. The small spatial overlap between HOMO and LUMO of **2** results in strong intramolecular charge-transfer character for S_1_ transition, thus renders exceptional bathochromic shift of absorption with low molar absorptivity.

### 2.5. Electrochemical Measurements

The results of the cyclic voltammetry (CV) measurements of all 8 compounds can be found in the overview in (SI Figure S4). As the oxidation onset (HOMO) could not be reached in all cases, in those cases, only the reduction CV is shown.

With the exception of the naphthyl-derivatives **3** and **4**, the measurements are in good agreement comparing the butyl decorated versions with the ethyl-adamantyl decorated ones. Like many organic compounds with carbonyl groups, the phenanthrene-derivatives **5** and **6** showed an orange color on the electrode upon reduction. Interestingly, the pyrene-derivatives **7** and **8** showed a similar behavior upon oxidation, which might be explained with a partially reversible oxidation character.

The results of the CV measurements and HOMO-LUMO determination of the compounds **1** to **8** are summarized in Table 3. It should be stressed that the flavins under investigation possess LUMO orbitals of considerably low energies ranging from (−3.72) to (−4.20) eV, similar to their NH-free flavin analogues. The low LUMO levels are particularly ascribed to the strong electron-accepting character of the conjugated pyrazine and uracil moieties in the structure of flavin and might be favorable for the applications of the materials for catalytic reactions like hydrogen evolution reaction in water or oxygen evolution.

### 2.6. Thin Film Microscopy

**Figure 6:**
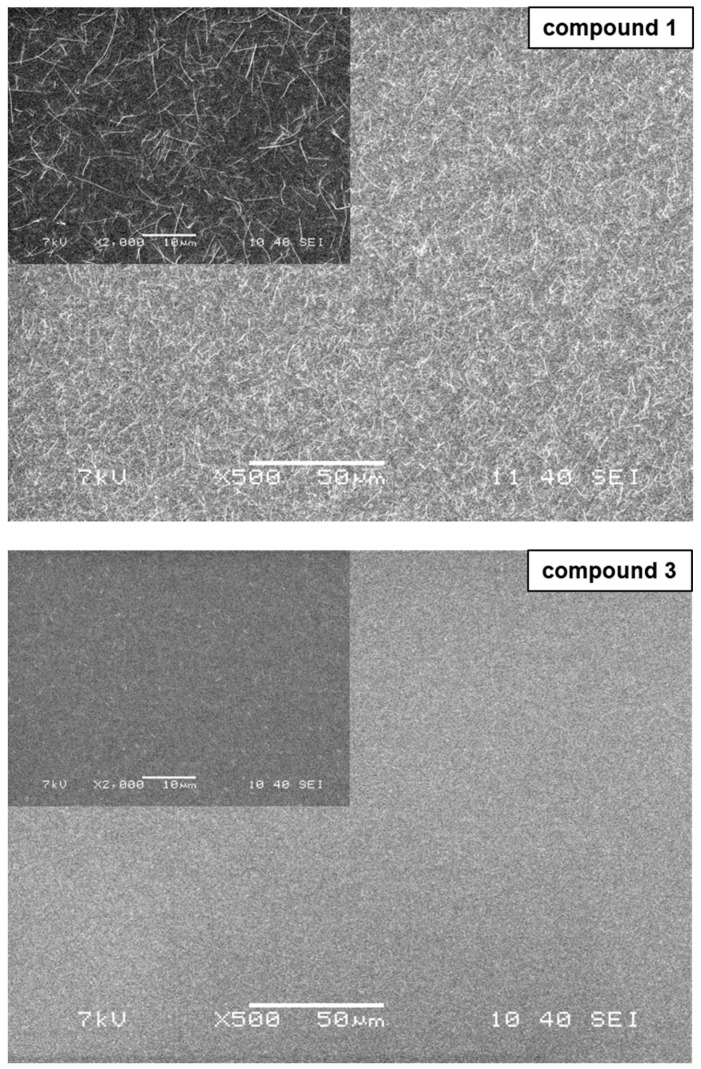
SEM pictures of the thin films surface of compounds **1** and **3** with a magnification of 500× and 2000×.

Scanning electron microscopy (SEM) was performed to investigate the thin film microscopy of 150 nm thick vacuum evaporated films. Almost all eight compounds show a quite uniform and homogeneous surface up to a sub-micrometer range, which is beneficial for the application in organic electronic devices. The SEM images of butyl-substituted benzene-flavin compound **1** were a slight exception to this, as a slightly rougher surface caused by string-like structures was observed (compare e.g., SEM records of **1** and **3** in Figure 6). Although also the compounds **2**–**7** showed some slight surface defects, most probably caused by aggregation, the several micrometer long structures of compound **1** were exceptional. The SEMs of all of the evaporated thin layers can be found in (SI Figure S5).

### 2.7. Thin Film Aging

In the importance of potential thin-film device lifetime, aging of the vacuum deposited layers was being investigated in the period of two months. Between the measurements, substrates were stored at the dark place under ambient conditions. As a first hint, the absorption and emission spectra were measured and compared. Figure S6 shows a comparison with the most significant change in the spectra of compound **1** and **2**, which indicated a change in the morphology. As proof, SEM scans were done for thin films of these flavin derivatives (Figure S7). Thin films of the other materials were rather unchanged with the same properties as after deposition.

## 3. Conclusions

The present paper deals with the synthetic approach and comprehensive physico-chemical characterization, including thermal properties, optics, charge transport, DFT modelling, electrochemistry, and morphological studies of the thin films of the novel soluble *N*,*N*′-alkylated flavin-inspired derivatives. The materials possess chemical versatility and high thermal stability reaching 475 °C, which suggests their promising future potential as building blocks in supramolecular systems or copolymers. Extension of the conjugated system of the alloxazine moiety bathochromically shifts the absorption and emission spectra both in thin film and solution independent of the attached alkyl chain. The energies of FMO determined using cyclic voltammetry showed the LUMO orbitals of noticeably low energies ranging from (−3.72) to (−4.20) eV. The SEM investigations of vacuum deposited films demonstrated rather homogeneous and smooth surface of the films on the sub-micrometer scale, being mostly stable over two-month period, demonstrating the stability of the flavins in thin films. Aforementioned application versatility, processability, and photocatalytic activity of the novel alkylated flavins, and the promising optical and electrochemical behavior could infer their application e.g., in the photocatalytic oxygen and hydrogen evolution or as effective redox centers in biomimetic energy storage.

## 4. Experimental Section

### 4.1. Materials and Synthesis

All solvents and reagents were obtained commercially and used as received unless stated otherwise. All moisture-sensitive reactions were performed in dry flasks fitted with glass stoppers or rubber septa under a positive pressure of argon. Air- and moisture-sensitive liquids and solutions were transferred by syringe or stainless-steel cannula. Anhydrous Na_2_SO_4_ was used to dry organic solutions during workup, and evaporation of the solvents was performed under reduced pressure using a rotary evaporator. Flash column chromatography was performed using 220–440 mesh silica gel. Thin-layer chromatography was conducted on TLC plates Supelco 60 with fluorescence indicator 254 nm. Spots were observed under UV irradiation (254 nm or 354 nm). ^1^H- and ^13^C-NMR spectra were recorded in CDCl_3_ using Bruker Avance III 500 MHz spectrometer with working frequencies of 500 MHz and 125 MHz, respectively, at 30 °C. Chemical shifts are expressed in parts per million (δ scale) downfield from tetramethylsilane and are referenced to residual protium in the NMR solvent (CHCl_3_: δ7.25 ppm). Coupling constants (*J*) are given in Hz with coupling expressed as s—singlet, bs—broad singlet, d—dublet, dd—doublet of doublet, t—triplet, tdd—doublet of triplet of doublets, ddd—doublet of doublet of doublets, m—multiplet. Elemental analysis was performed using EuroEA3000 Elemental Analyzer. Melting points were determined using Kofler apparatus with microscope Nagema PHMK 05. Thermogravimetric analysis was performed using TA Instruments TGA Q50 with nitrogen as the carrier gas.

### 4.2. General Procedure for the Synthesis of N,N′-Dialkylated Flavins

Non-alkylated flavin (1.60 mmol, 1 eq.) was dispersed in anhydrous DMF (25 mL) under argon atmosphere. Subsequently, potassium carbonate or cesium carbonate (6.40 mmol, 4 eq.) and corresponding alkylbromide (6.40 mmol, 4 eq.) were added and the mixture was heated to 60 °C for 16 h. Afterwards, the reaction mixture was cooled to room temperature and poured to water (250 mL). The suspension was sonicated for 30 min and filtered. The resulting solid was washed with cold methanol (20 mL) and dried under reduced pressure. The crude product was adsorbed to silica gel and purified by silica gel column chromatography and subsequent digestion from boiling alcohol to yield target molecules.

### 4.3. 1,3-Dibutylbenzo[g]pteridine-2,4(1H,3H)-dione (***1***)

Compound **1** was prepared according to the General procedure starting with Compound A (0.23 g, 1.08 mmol, 1 eq.), K_2_CO_3_ (0.60 g, 4.32 mmol, 4 eq.), and 1-bromobutane (0.59 g, 4.32 mmol, 4 eq.) providing flavin **1** as light green–white in yield 0.23 g (65%) after purification of the crude product by column chromatography using petroleum ether/ethyl acetate (80/20) as the elution mixture and subsequent digestion from boiling ethanol.

M.P. 135–137 °C. ^1^H-NMR (CDCl_3_, TMS, 500 MHz): δ 8.32 (dd, *J* = 8.6, 1.4 Hz, 1H), 8.02 (dd, *J* = 8.6, 1.4 Hz, 1H), 7.88 (ddd, *J* = 8.6, 6.8, 1.4 Hz, 1H), 7.74 (ddd, *J* = 8.6, 6.8, 1.4 Hz, 1H), 4.50–4.40 (m, 2H), 4.23–4.14 (m, 2H), 1.84–1.69 (m, 4H), 1.53–1.39 (m, 4H), 1.04–0.95 (m, 6H). ^13^C-NMR (CDCl_3_, TMS, 126 MHz): δ 13.9, 14.0, 20.3, 20.3, 29.8, 30.0, 42.6, 42.7, 128.0, 129.1, 130.0, 130.9, 133.8, 140.2, 143.6, 145.3, 150.4, 159.7. Elemental analysis calcd (%) for C_18_H_22_N_4_O_2_: C 66.24, H 6.79, N 17.17; found: C 66.22, H 6.81 N 17.19.

### 4.4. 1,3-Bis(2-(-adamantan-1-yl)ethyl)benzo[g]pteridine-2,4(1H,3H)- dione (***2***)

Compound **2** was prepared according to the General procedure starting with Compound A (0.30 g, 1.40 mmol, 1 eq.), Cs_2_CO_3_ (2.28 g, 7.00 mmol, 5 eq.), and 1-(2-bromoethyl)adamantane (1.70 g, 7.00 mmol, 5 eq.) providing flavin **2** as light green–white in yield 0.35 g (47%) after purification of the crude product by two cycles of column chromatography using petroleum ether/ethyl acetate (80/20) as the elution mixture and subsequent digestion from boiling ethanol.

M.P. 251–252 °C. ^1^H-NMR (CDCl_3_, TMS): δ 8.31 (dd, *J* = 8.5, 1.3 Hz, 1H), 8.01 (dd, *J* = 8.5, 1.3 Hz, 1H), 7.89–7.85 (m, 1H), 7.77–7.70 (m, 1H), 4.57–4.40 (m, 2H), 4.24–4.17 (m, 2H), 2.05–1.97 (m, 6H), 1.62–1.80 (m, 24H), 1.80 (m, 24H), 1.58–1.44 (m, 4H). Elemental analysis calcd (%) for C_34_H_42_N_4_O_2_: C 75.80, H 7.86, N 10.40; found: C 75.82, H 7.84, N 10.42.

### 4.5. 1,3-Dibutylnaphtho[2,3-g]pteridine-2,4(1H,3H)-dione (***3***)

Compound **3** was prepared according to the General procedure starting with Compound B (0.23 g, 1.42 mmol, 1 eq.), K_2_CO_3_ (0.98 g, 7.11 mmol, 5 eq.), and 1-bromobutane (0.98 g, 7.11 mmol, 5 eq.) providing flavin **3** as bright red solid in yield 0.15 g (28%) after purification of the crude product by two cycles of column chromatography using dichloromethane as the eluent and subsequent digestion from boiling ethanol.

M.P. 210–211 °C. ^1^H-NMR (CDCl_3_, TMS, 5,00 MHz): δ 8.93 (s, 1H), 8.52 (s, 1H), 8.11 (d, *J* = 8.1 Hz, 1H), 8.05 (d, *J* = 8.1 Hz, 1H), 7.61 (ddd, *J* = 8.1, 6.6, 1.3 Hz, 1H), 7.55 (ddd, *J* = 8.1, 6.6, 1.3 Hz, 1H), 4.50–4.44 (m, 2H), 4.24–4.15 (m, 2H), 1.87–1.79 (m, 2H), 1.79–1.72 (m, 2H), 1.55–1.42 (m, 4H), 1.04 (t, *J* = 7.4 Hz, 3H), 0.99 (t, *J* = 7.4 Hz, 3H). ^13^C-NMR (CDCl_3_, TMS, 126 MHz): δ 13.9, 14.0, 20.3, 20.3, 29.8, 30.1, 42.6, 42.8, 125.4, 126.7, 128.1, 128.7, 129.3, 130.6, 131.7, 133.3, 136.5, 137.1, 138.9, 144.3, 150.3, 159.5. Elemental analysis calcd (%) for C_22_H_24_N_4_O_2_: C 70.19, H 6.43, N 14.88; found: C 70.17, H 6.41, N 14.91.

### 4.6. 1,3-Bis(2-(adamantan-1-yl)ethyl)naphtho[2,3-g]pteridine2,4(1H,3H)-dione (***4***)

Compound **4** was prepared according to the General procedure starting with Compound B (0.30 g, 1.90 mmol, 1 eq.), K_2_CO_3_ (1.31 g, 9.48 mmol, 5 eq.), and 1-(2-bromoethyl)adamantane (2.31 g, 9.48 mmol, 5 eq.) providing flavin **4** as yellowish solid in yield 0.35 g (31%) after purification of the crude product by column chromatography using dichloromethane as the eluent and subsequent digestion from boiling ethanol.

M.P. 308-309 °C. ^1^H-NMR (CDCl_3_, TMS): δ 8.95 (s, 1H), 8.54 (s, 1H), 8.13 (d, *J* = 8.5 Hz, 1H), 8.09 (d, *J* = 8.5 Hz, 1H), 7.66–7.59 (m, 1H), 7.59–7.53 (m, 1H), 4.57–4.46 (m, 2H), 4.26–4.16 (m, 2H), 2.05 (br s, 3H), 2.00 (br s, 3H), 1.82–1.64 (m, 24H), 1.61–1.56 (m, 2H), 1.54–1.47 (m, 2H). Elemental analysis calcd (%) for C_38_H_44_N_4_O_2_: C 77.52, H 7.53, N 9.52; found: C 77.55, H 7.51, N 9.50.

### 4.7. 10,12-dibutylphenanthro[9,10-g]pteridine-11,13(10H,12H)-dione (***5***)

Compound **5** was prepared according to the General procedure starting with Compound C (0.25 g, 0.80 mmol, 1 eq.), K_2_CO_3_ (0.55 g, 3.98 mmol, 5 eq.), and 1-bromobutane (0.55 g, 3.98 mmol, 5 eq.) providing flavin **5** as yellow solid in yield 0.39 g (85%) after purification of the crude product by column chromatography using dichloromethane as the eluent and subsequent digestion from boiling methanol.

M.P. 197–198 °C. ^1^H-NMR (CDCl_3_, TMS): δ 9.48 (dd, *J* = 7.7, 1.2 Hz, 1H), 9.21 (dd, *J* = 7.7, 1.2 Hz, 1H), 8.34 (dd, *J* = 7.7, 1.2 Hz, 1H), 8.28 (dd, *J* = 7.7, 1.2 Hz, 1H), 8.13–8.01 (m, 4H), 4.59–4.54 (m, 2H), 4.27–4.23 (m, 2H), 1.96–1.89 (m, 2H), 1.85–1.78 (m, 2H), 1.65–1.54 (m, 2H), 1.53–1.46 (m, 2H), 1.08 (t, *J* = 7.4 Hz, 3H), 1.03 (t, *J* = 7.4 Hz, 3H). Elemental analysis calcd (%) for C_26_H_26_N_4_O_2_: C 73.22, H 6.14, N 13.14; found: C 73.25, H 6.17, N 13.11.

### 4.8. 10,12-Bis(2-(adamantan-1-yl)ethyl)phenanthro[9,10-g]pteridine11,13(10H,12H)-dione (***6***)

Compound **6** was prepared according to the General procedure starting with Compound C (0.24 g, 0.75 mmol, 1 eq.), Cs_2_CO_3_ (1.23 g, 3.77 mmol, 5 eq.), and 1-(2-bromoethyl)adamantane (0.73 g, 3.02 mmol, 4 eq.) providing flavin **6** as yellow solid in yield 0.11 g (23%) after purification of the crude product by two cycles of column chromatography using gradient elution by mixture petroleum ether/dichloromethane (50/50 → 0/100) as the eluent and subsequent digestion from boiling methanol.

M.P. > 350 °C. ^1^H-NMR (CDCl_3_, TMS): δ 9.53 (d, *J* = 7.7 Hz, 1H), 9.32 (d, *J* = 7.7 Hz, 1H), 8.36 (d, *J* = 7.7 Hz, 1H), 8.30 (d, *J* = 7.7 Hz, 1H), 8.14–8.05 (m, 4H), 4.66–4.59 (m, 2H), 4.30–4.24 (m, 2H), 2.09 (s, 3H), 2.02 (s, 3H), 1.84–1.62 (m, 24H), 1.60–1.50 (m, 4H). Elemental analysis calcd (%) for C_42_H_48_N_4_O_2_: C 78.71, H 7.55, N 8.74; found: C 78.73, H 7.52, N 8.79.

### 4.9. 10,12-Dibutyl-5a^1^,10-dihydropyreno[4,5-g]pteridine11,13(3a^1^H,12H)-dione (***7***)

Compound **7** was prepared according to the General procedure starting with Compound D (0.34 g, 1.01 mmol, 1 eq.), K_2_CO_3_ (0.69 g, 5.02 mmol, 5 eq.), and 1-bromobutane (0.55 g, 4.02 mmol, 4 eq.) providing flavin **7** as yellow-green solid in yield 0.18 g (41%) after purification of the crude product by two cycles of column chromatography using gradient elution by mixture petroleum ether/dichloromethane (50/50 → 0/100) as the eluent and subsequent digestion from boiling methanol.

M.P. 240–241 °C. ^1^H-NMR (CDCl_3_, TMS): δ 9.30 (dd, *J* = 7.9, 1.4 Hz, 1H), 9.07 (dd, *J* = 8.2, 1.4 Hz, 1H), 8.62 (d, *J* = 8.2 Hz, 1H), 8.58 (dd, *J* = 8.2, 1.4 Hz, 1H), 7.86 (ddd, *J* = 8.2, 7.0, 1.4 Hz, 1H), 7.81–7.72 (m, 3H), 4.59–4.52 (m, 2H), 4.27–4.19 (m, 2H), 1.89 (p, 2H), 1.79 (p, 2H), 1.61–1.44 (m, 4H), 1.06 (t, *J* = 7.4 Hz, 3H), 1.01 (t, *J* = 7.4 Hz, 3H). Elemental analysis calcd (%) for C_28_H_28_N_4_O_2_: C 74.31, H 6.24, N 12.38; found: C 74.29, H 6.21, N 12.35.

### 4.10. 10,12-Bis(2-(adamantan-1-yl)ethyl)-5a^1^,10-dihydropyreno[4,5-g]pteridine-11,13(3a^1^H,12H)-dione (***8***)

Compound **8** was prepared according to the General procedure starting with Compound D (0.27 g, 0.78 mmol, 1 eq.), Cs_2_CO_3_ (1.27 g, 3.92 mmol, 5 eq.), and 1-(2-bromoethyl)adamantane (0.95 g, 3.96 mmol, 5 eq.) providing flavin **8** as yellow-green solid in yield 0.29 g (55%) after purification of the crude product by column chromatography using gradient elution by mixture petroleum ether/dichloromethane (50/50 → 0/100) as the eluent and subsequent digestion from boiling methanol.

M.P. > 350 °C. ^1^H-NMR (CDCl_3_, TMS, 500 MHz): δ 9.28 (dd, *J* = 8.1, 1.6 Hz, 1H), 9.01 (dd, *J* = 8.1, 1.6 Hz, 1H), 8.60 (d, *J* = 8.1 Hz, 1H), 8.56 (dd, *J* = 8.1, 1.6 Hz, 1H), 7.84 (ddd, *J* = 8.1, 7.0, 1.6 Hz, 1H), 7.81–7.68 (m, 3H), 4.53–4.44 (m, 2H), 4.28–4.17 (m, 2H), 2.06 (t, *J* = 3.2 Hz, 3H), 2.02 (t, *J* = 3.2 Hz, 3H), 1.82–1.66 (m, 24H), 1.62–1.51 (m, 4H). Elemental analysis calcd (%) for C_44_H_48_N_4_O_2_: C 79.48, H 7.28, N 8.43; found: C 79.45, H 7.29, N 8.45.

## Figures and Tables

**Table 1:** Data from TGA measurements and melting points for the alkylated flavins (**1**–**8**) and the unsubstituted derivatives (**A**–**D**). The decomposition temperature is reported as the temperature at the steepest point (1st derivation) of the TGA curve (see SI).

Flavin	1	2	3	4	5	6	7	8	A	B	C	D
Decomposition temperature (°C)	274	380	338	422	385	475	364	475	377	393	398	421
Melting point (°C)	135–137	251–252	210–211	308–309	197–198	>350	240–241	>350	>350	>350	>350	>350

**Table 2:** The optical parameters of the compounds.

	Solution		Solid State		
#	abs_max_^*^/nm	em_max_/nm	abs_max_^*^/nm	em_max_/nm	*ϕ_F_*
1	385	423,442	386	434	0.01
2	386	424,442	385	438	0.01
3	398	589	397	579	0.02
4	398	584	397	597	0.02
5	415	431	420	480	0.02
6	416	430	419	475,495	0.02
7	455	497	466	514	0.02
8	456	494	457	528	0.02

abs_max_^*^—absorption maximum (closest to the emission spectrum); em_max_—emission maximum; *ϕ_F_*—photoluminescence quantum yield.

**Table 3:** Oxidation and reduction potentials and frontier molecular orbitals (FMO) levels obtained by the electrochemical measurements.

Compound	LUMO/V vs. SHE	HOMO/V vs. SHE	LUMO/eV	HOMO/eV	E_g(echem)_/eV
1	−0.91	-	−3.84	-	-
2	−0.86	-	−3.89	-	-
3	−0.72	+1.79	−4.03	−6.54	2.51
4	−0.55	+1.87	−4.20	−6.62	2.42
5	−1.03	+1.71	−3.72	−6.46	2.74
6	−1.00	-	−3.75	-	-
7	−0.98	+1.76	−3.77	−6.51	2.74
8	−0.97	+1.78	−3.78	−6.53	2.75

## Data Availability

The data presented in this study are available in article and Supplementary Materials.

## Supplementary Materials

The following are available online, Figure S1: TGA records of the compounds **1**–**8**, Figure S2: Depiction of FMO energy levels and Eg calculated using Gaussian 09 DFT B3LYP/6-31G** CPCM=CHCl3, Figure S3: Calculated absorption spectra using Gaussian 09 DFT B3LYP/6- 31G** CPCM=CHCl3. Gaussian envelope of 0.2 eV, Figure S4: Cyclic voltammograms of the compounds **1**–**8**, Figure S5: SEM pictures of the flavin thin films surface with a magnification of 500× and 6000×, Figure S6: The comparison of the absorption and emission spectra of flavin derivatives measured before and after the period of two months, Figure S7: The comparison of the SEM scans of flavin derivatives measured before and after the period of two months with magnification of 2000×, Table S1: Energies of FMO and E_g_ calculated using Gaussian 09 DFT B3LYP/6-31G** CPCM=CHCl_3_, Table S2: Calculated characteristics of S_0_→S_1_ transitions using Gaussian 09 DFT B3LYP/6- 31G** CPCM=CHCl_3_. Gaussian envelope of 0.2 eV, Table S3: Summary of the hole field-effect mobilities.
