# Supplementary material for: Tunable Properties of Nature-Inspired N,N′-Alkylated Riboflavin Semiconductors
Source: Molecules. 2020 Dec 23;26(1):27. doi: 10.3390/molecules26010027 (PMC7793104; doi:10.3390/molecules26010027)
Supplement: Supplementary file 1 [file molecules-26-00027-s001.pdf]

Supporting Information

Tunable properties of Nature-Inspired N,N′-Alkylated Riboflavin Semiconductors

Jan Richtar[a], Lucia Ivanova[a], Dong Ryeol Whang[b], Cigdem Yumusak[b], Dominik Wielend[b], Martin Weiter[a], Markus Clark Scharber[b], Alexander Kovalenko[a], Niyazi Serdar Sariciftci[b], Jozef Krajcovic[a]*

[a] *Brno University of Technology, Faculty of Chemistry, Materials Research Centre, Purkyňova 118, 612 00 Brno, Czech Republic.*
Email: krajcovic@fch.vut.cz
[b] *Linz Institute for Organic Solar Cells (LIOS), Physical Chemistry, Johannes Kepler University Linz, Altenbergerstraße 69, 4040 Linz, Austria.*

Contents

Preparation of the substrates........1
Film deposition........1
Optical measurements........1
Thermogravimetric analysis data........3
DFT........11
Field-effect mobilities........13
Cyclic voltammograms........14
SEM records........17
Thin-film aging........19
References........21

Preparation of the substrates

Glass and ITO coated glass substrates were cleaned with the same procedure in an ultrasonic bath of detergent and deionized water, acetone and isopropyl alcohol (IPA).

Film deposition

Physical vapor deposition technique was used for the fabrication of thin films for optical measurements, scanning electron microscopy and organic field-effect transistors (OFETs). For this purpose, custom-built organic evaporation system allowing precise thickness rate control from Vaksis R&D and Engineering was used. The process of evaporation was performed under vacuum (10^{-6} mbar) with a rate of 0.2 Å/s. The thickness of the deposited layers was verified by measurements on a Dektak XT mechanical profilometer (Bruker).

Optical measurements

Ultraviolet-Visible (UV-Vis) and fluorescence spectroscopy were used to determine the optical properties of flavin derivatives. For measurement of the optical properties of the flavins in the solid-

state, samples were prepared by physical vapour deposition on the glass slices. The thickness of the flavin films was roughly100 nm.

To measure the spectra of the solutions including evaluation of molar absorption coefficient of each material a set of solutions in chloroform (optical quality) was prepared with the concentrations of 0.01, 0.015, 0.02 and 0.025 mM. Measurements were performed using quartz cuvettes with a path length of 10.00 mm. Molar absorption coefficient ε of the whole spectrum of each molecule was calculated according to the Beer-Lambert law for absorbance.[1] All the optical measurements were done under ambient conditions.

The UV-Vis absorption spectroscopy was performed with Lambda1050 UV/Vis/NIR spectrometer (PerkinElmer). Fluorescence spectra were obtained with fluorimeter from Photon Technology International. Photoluminescence quantum yields (PLQY) of the thin films was determined using an absolute photoluminescence quantum yield spectrometer (Hamamatsu C9920-02) equipped with an integrating sphere, a xenon-lamp monochromator unit for photoexcitation and a photonic multichannel analyser for detection.

Thermogravimetric analysis data

Figure S1: TGA records of the compounds **1-8**.

Compound 1

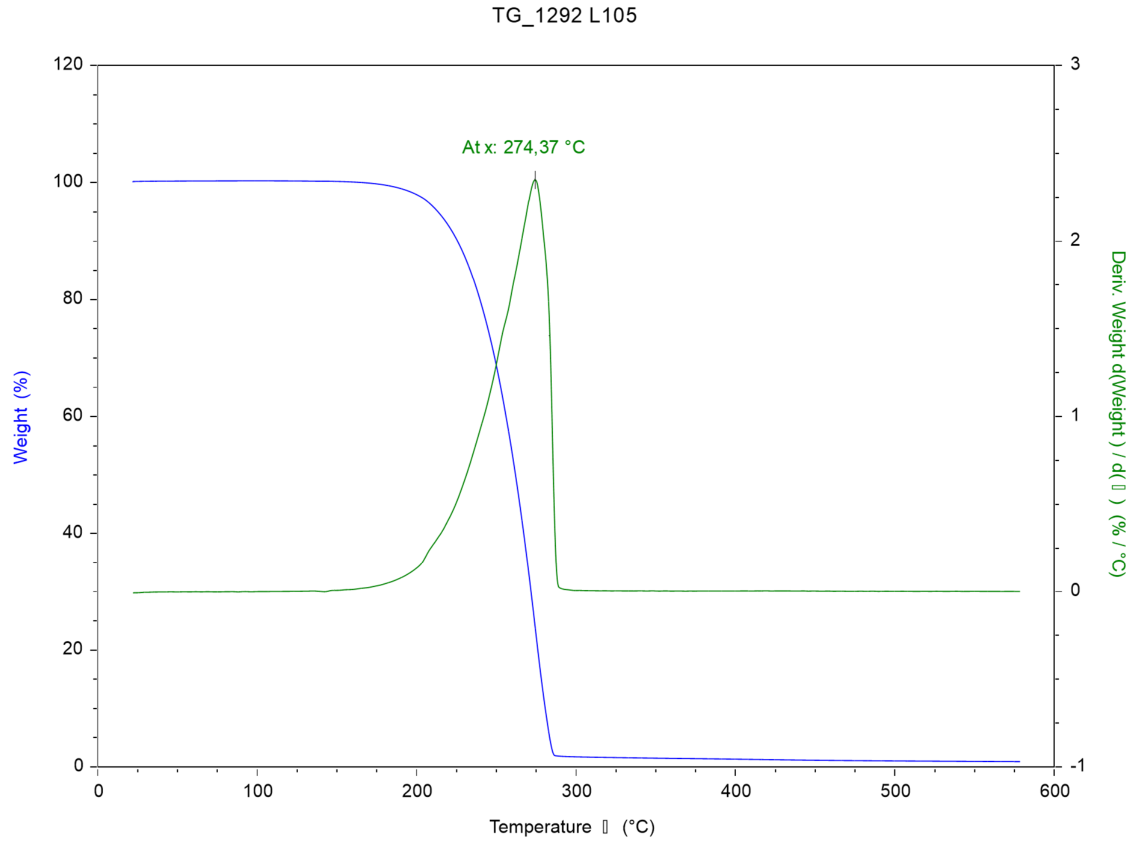

Compound 2

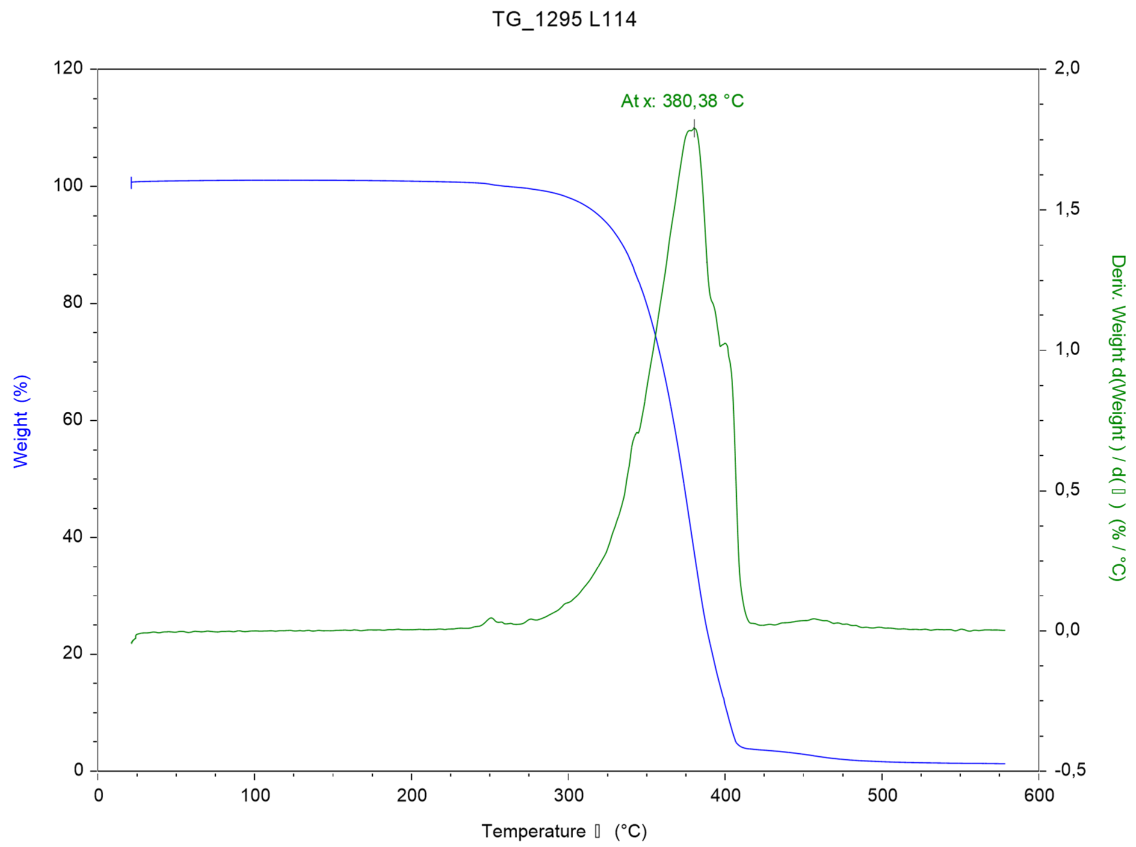

TG_1295 L114
At x: 380,38 °C
Weight (%)
Deriv. Weight d(Weight) / d( ) (% / °C)
Temperature (°C)
120
100
80
60
40
20
0
2,0
1,5
1,0
0,5
0,0
-0,5
0
100
200
300
400
500
600

Compound 3

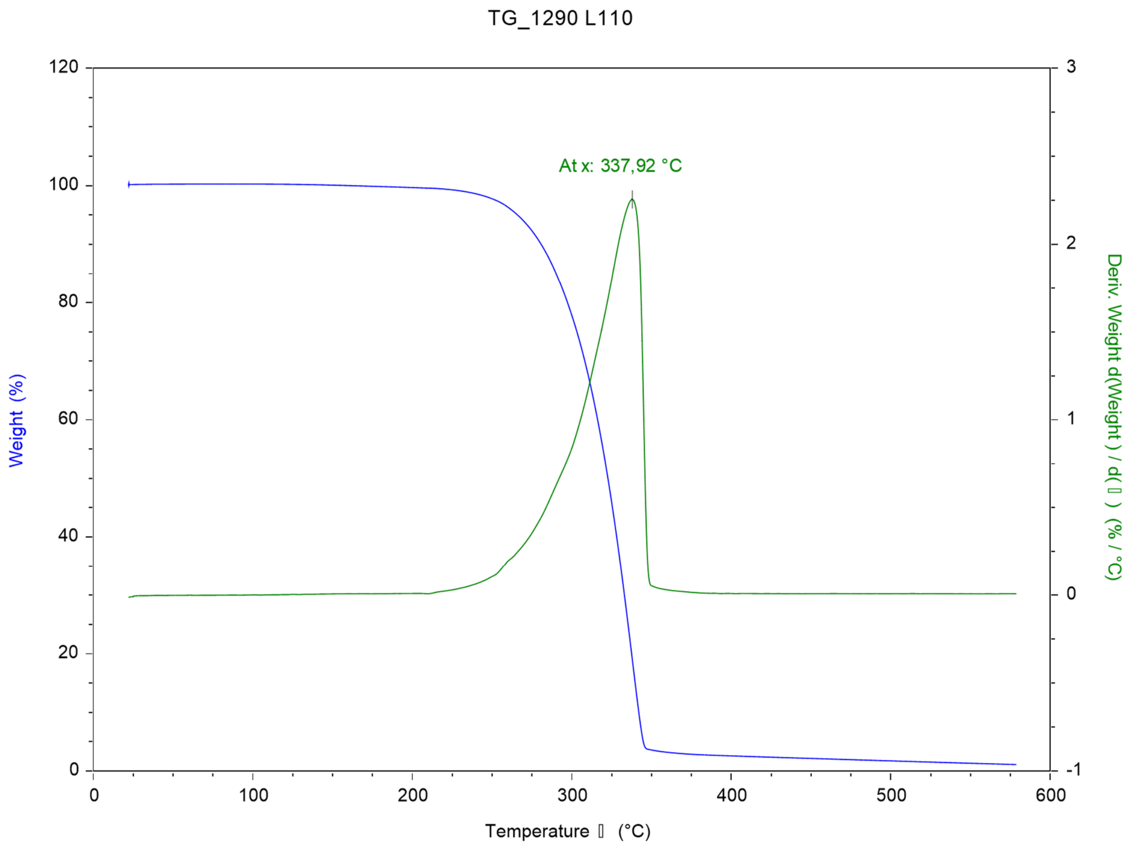

TG_1290 L110
At x: 337,92 °C
Weight (%)
Deriv. Weight d(Weight) / d( ) (% / °C)
Temperature (°C)
120
100
80
60
40
20
0
3
2
1
0
-1
0
100
200
300
400
500
600

Compound 4

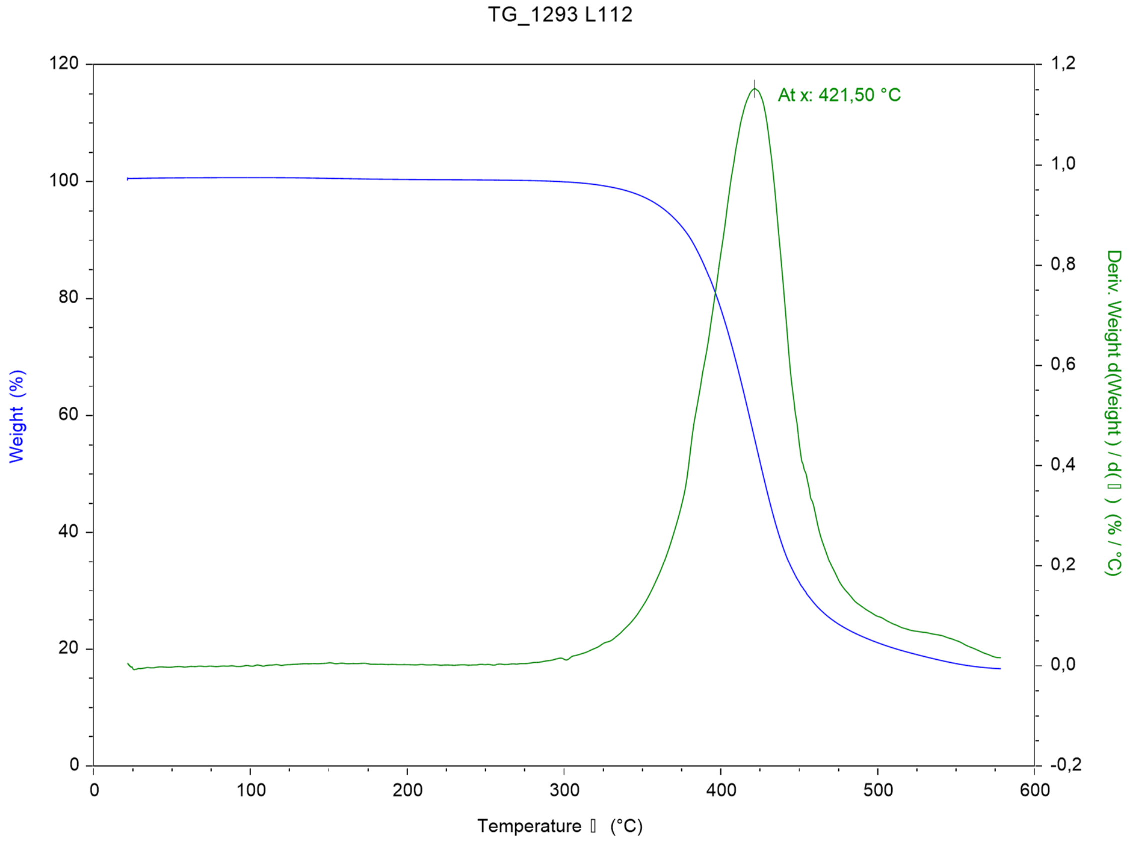

TG_1293 L112
At x: 421,50 °C
Weight (%)
Deriv. Weight d(Weight) / d( ) (% / °C)
Temperature (°C)
120
100
80
60
40
20
0
1,2
1,0
0,8
0,6
0,4
0,2
0,0
-0,2
0
100
200
300
400
500
600

Compound 5

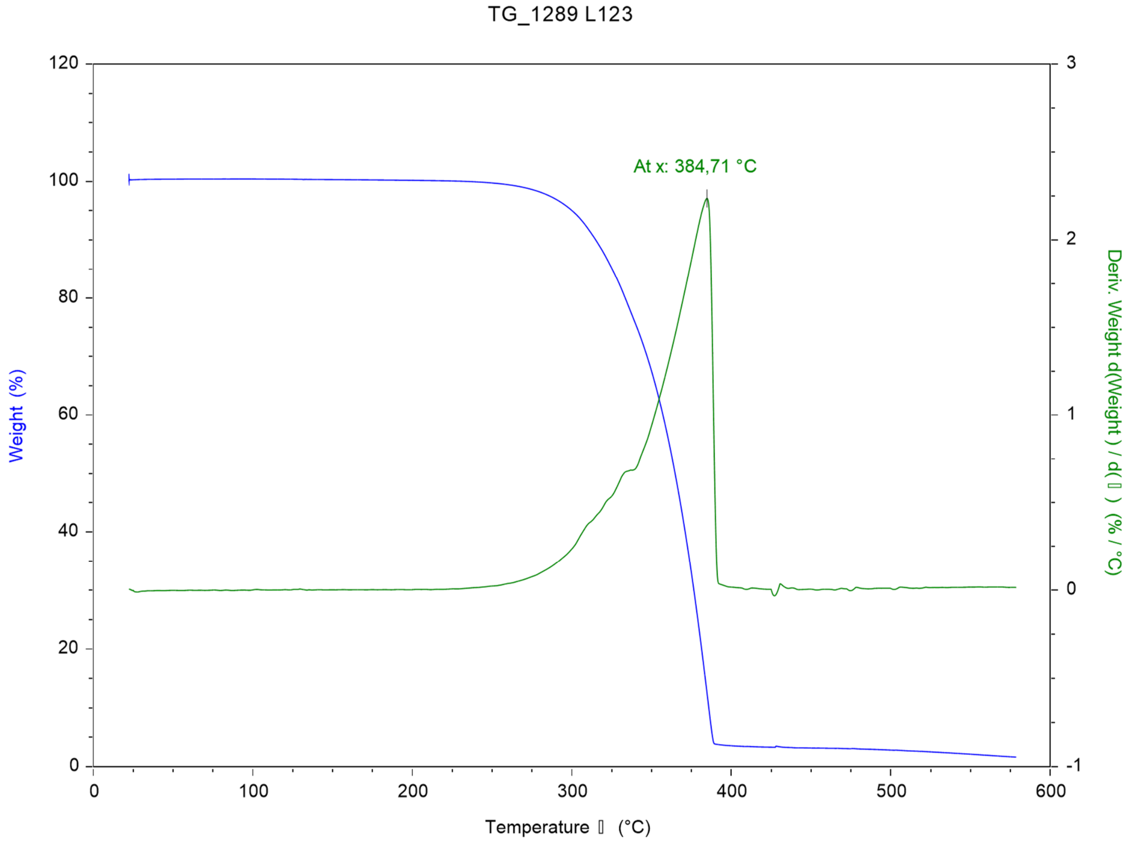

TG_1289 L123
At x: 384,71 °C
Weight (%)
Deriv. Weight d(Weight) / d( ) (% / °C)
Temperature (°C)
120
100
80
60
40
20
0
3
2
1
0
-1
0
100
200
300
400
500
600

Compound 6

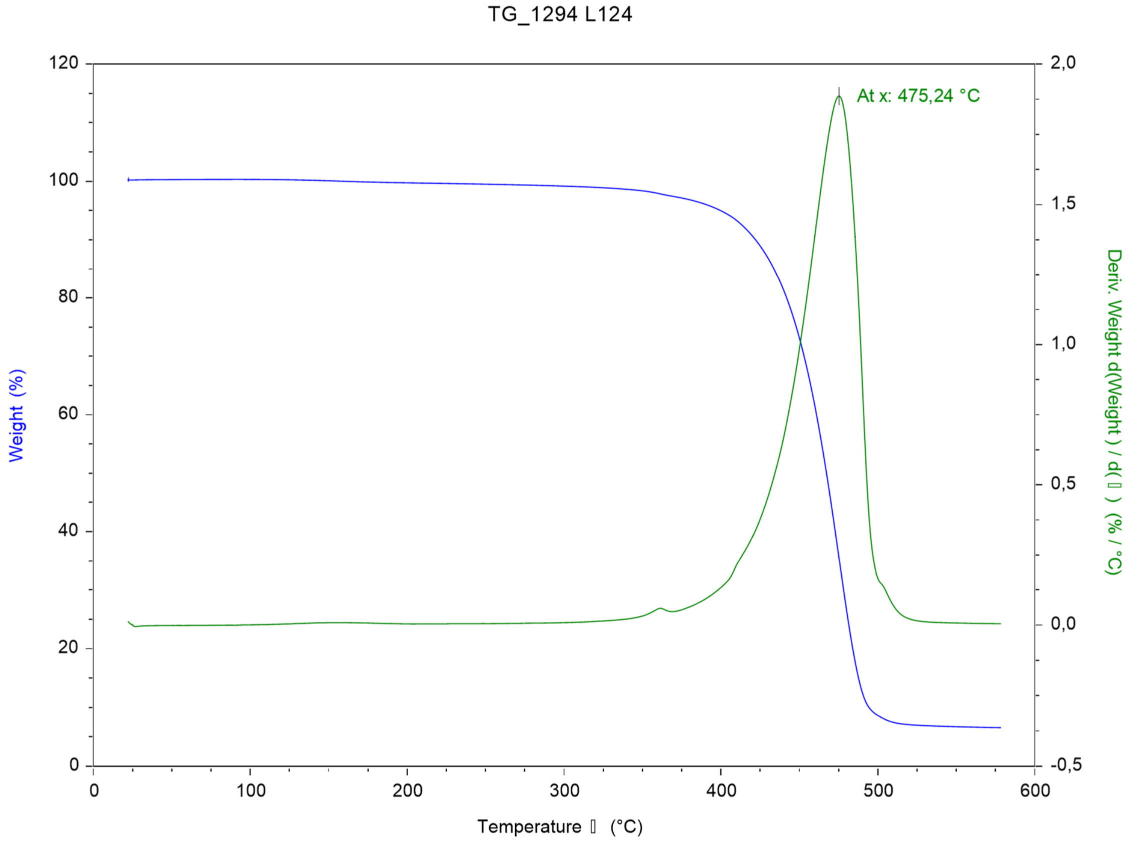

TG_1294 L124
At x: 475,24 °C
Weight (%)
Deriv. Weight d(Weight) / d( ) (% / °C)
Temperature (°C)
120
100
80
60
40
20
0
2,0
1,5
1,0
0,5
0,0
-0,5
0
100
200
300
400
500
600

Compound 7

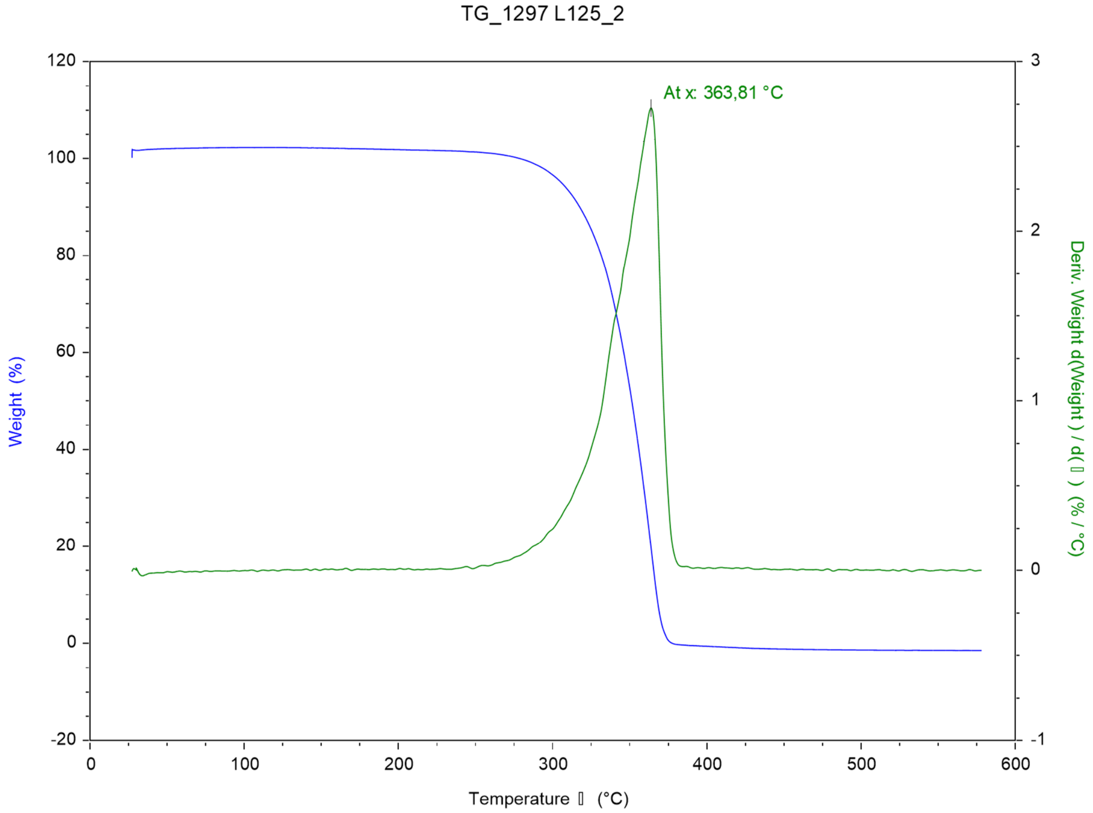

TG_1297 L125_2
At x: 363,81 °C
Weight (%)
Deriv. Weight d(Weight) / d( ) (% / °C)
Temperature (°C)
120
100
80
60
40
20
0
-20
3
2
1
0
-1
0
100
200
300
400
500
600

Compound 8

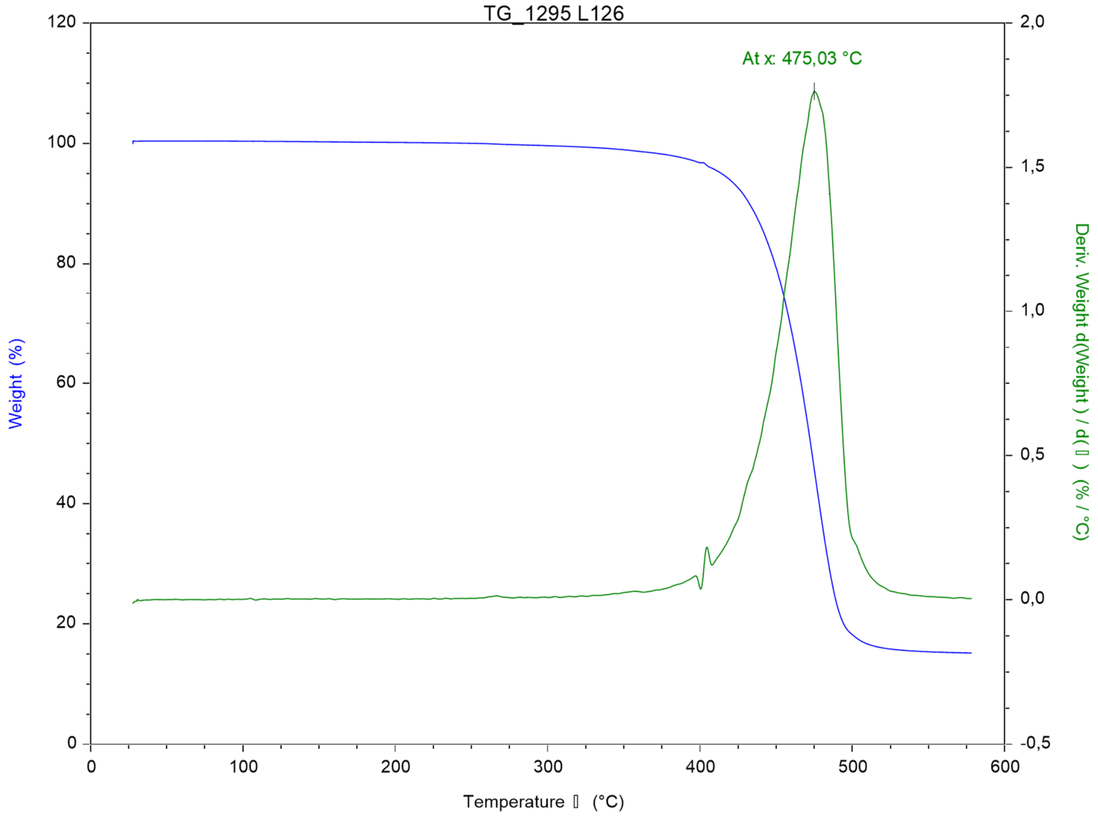

TG_1295 L126
At x: 475,03 °C
Weight (%)
Deriv. Weight d(Weight) / d( ) (% / °C)
Temperature (°C)
120
100
80
60
40
20
0
2,0
1,5
1,0
0,5
0,0
-0,5
0
100
200
300
400
500
600

DFT

Figure S2: Depiction of FMO energy levels and E_g calculated using Gaussian 09 DFT B3LYP/6-31G** CPCM=$CHCl_3$.

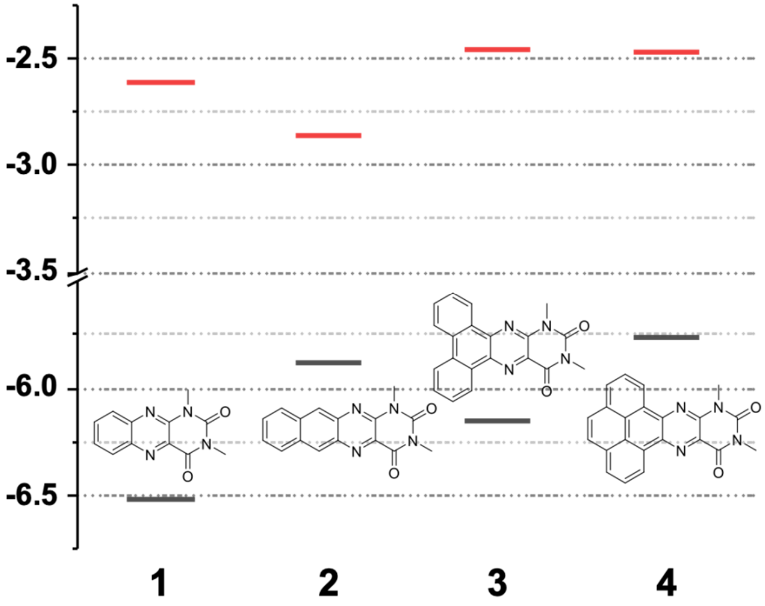

Table S1: Energies of FMO and E_g calculated using Gaussian 09 DFT B3LYP/6-31G** CPCM=$CHCl_3$.

entry	HOMO (eV)	LUMO (eV)	E_g (eV)
**1**	-6.53	-2.62	3.91
**2**	-5.89	-2.86	3.03
**3**	-6.16	-2.47	3.69
**4**	-5.76	-2.48	3.28

Figure S3: Calculated absorption spectra using Gaussian 09 DFT B3LYP/6- 31G** CPCM=$CHCl_3$. Gaussian envelope of 0.2 eV.

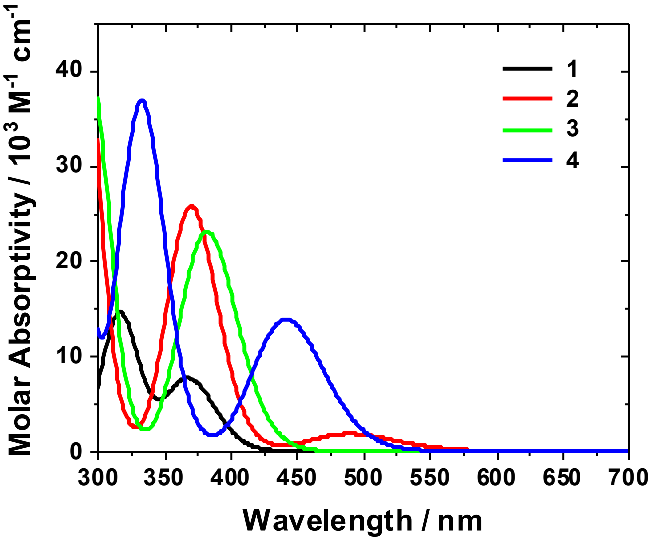

Table S2: Calculated characteristics of $S_0 \rightarrow S_1$ transitions using Gaussian 09 DFT B3LYP/6- 31G** CPCM=$CHCl_3$. Gaussian envelope of 0.2 eV.

entry	transition energy		*f*
	eV	nm	
**1**	3.36	369	0.111
**2**	2.53	490	0.027
**3**	3.19	389	0.189
**4**	2.80	442	0.205

Geometries of electronic ground states of the alloxazine derivatives were computed using the B3LYP level of density functional theory (DFT),[3,4] in which the 6-31G** basis set was applied for all atoms. Vertical transitions of the molecules were calculated at the time-dependent DFT (TD-DFT) using the conductor-like polarizable continuum model (CPCM)[5,6] to simulate solvent effect. All calculations were carried out using Gaussian 09.[7]

Field-effect mobilities

Table S3: Summary of the hole field-effect mobilities.

#	hole field-effect mobility μ_h /cm^2·V^{-1}·s^{-1}
**1**	N/A
**2**	N/A
**3**	N/A
**4**	N/A
**5**	$3{,}8\times10^{-8}$
**6**	$3{,}2\times10^{-8}$
**7**	$3{,}0\times10^{-8}$
**8**	$6{,}5\times10^{-8}$

OFETs were fabricated to determine field-effect charge carrier mobilities. Configuration of the transistors was bottom-gate/bottom-contact on heavily doped Si wafers (Fraunhofer IPMS). Substrates were cleaned by acetone, isopropanol and 18 MΩ water twice to enhance hydrophobicity. Subsequently, substrates were treated with O_2 plasma for 5 min at 100 W and hexamethyldisilazane (HMDS)[2] layer was formed by spin-coating (30 s, 2000 rpm/min). Consequently, 80 nm of the flavin derivative were vacuum deposited onto the treated silicon substrates by the procedure mentioned above.

Cyclic voltammograms

Figure S4: Cyclic voltammograms of the compounds **1-8.**

Compound 1

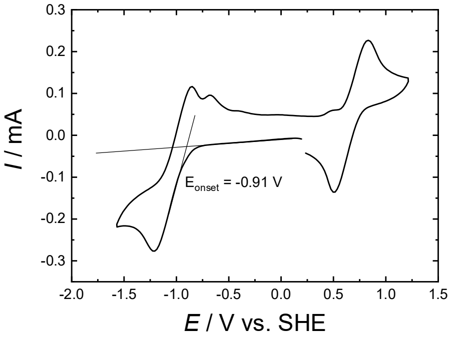

Compound 2

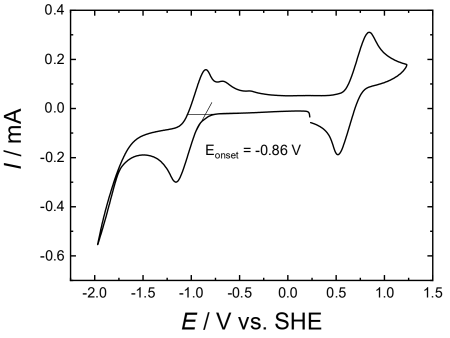

Compound 3

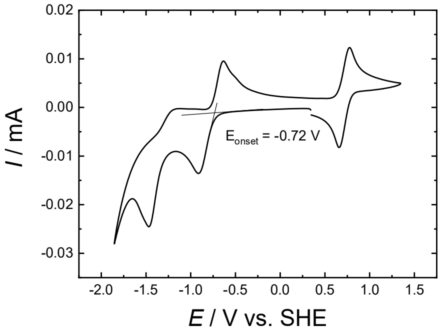

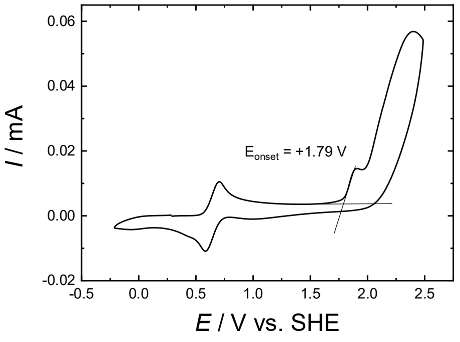

Compound 4

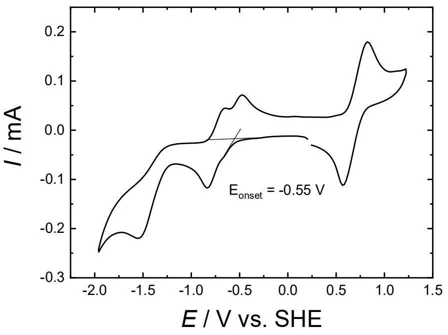

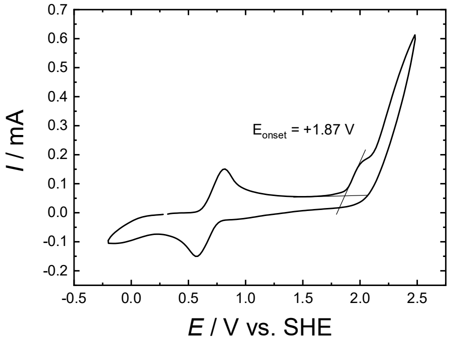

Compound 5

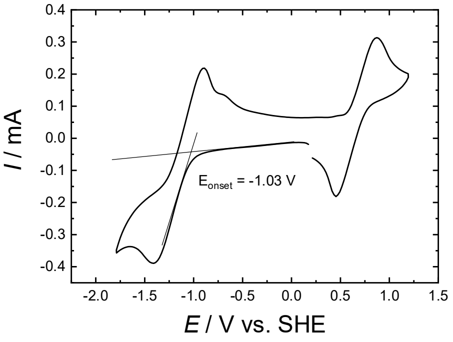

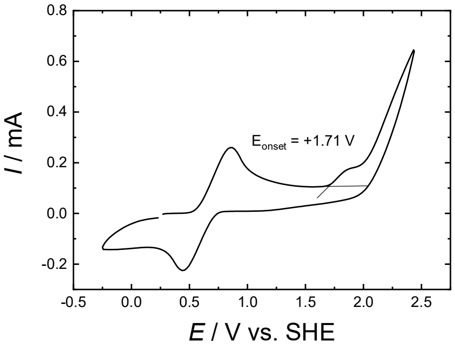

Compound 6

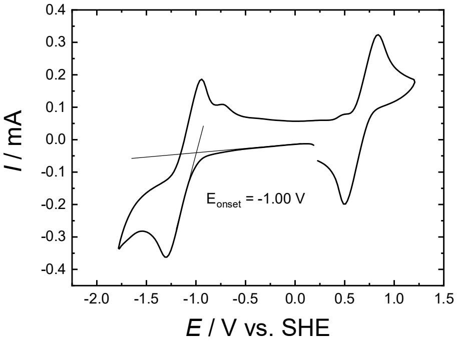

Compound 7

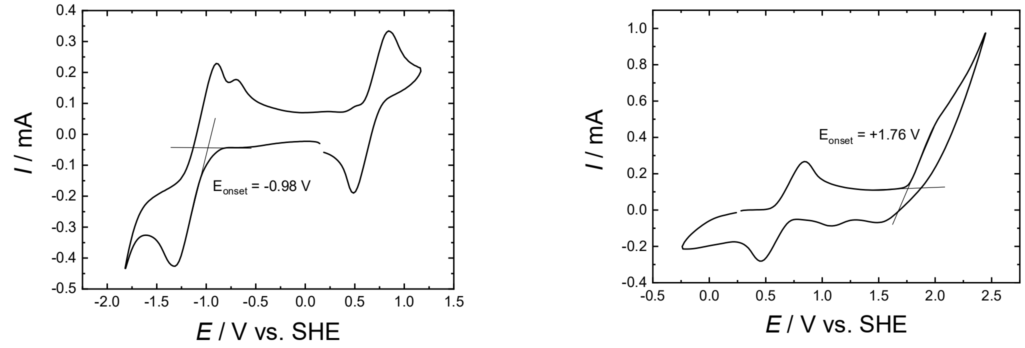

Compound 8

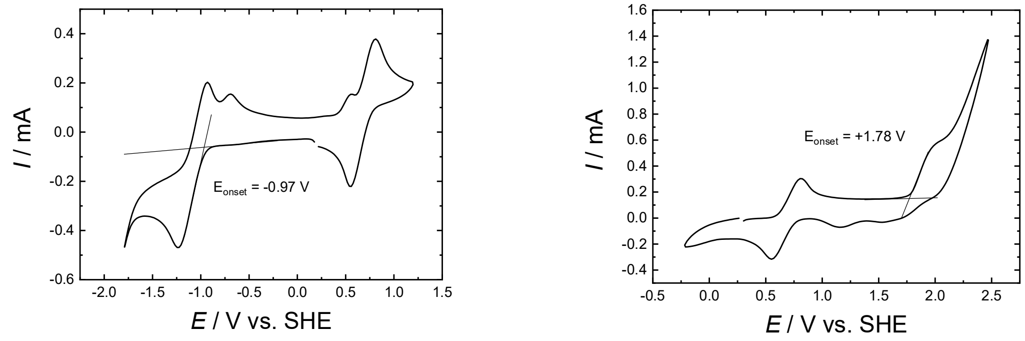

Cyclic voltammetry experiments were performed for all materials with an IPS-Jaissle Potentiostat/Galvanostat PGU10V-100mA. A one-compartment cell was used as electrochemical cell with a platinum working electrode (WE), a platinum counter electrode (CE) and an Ag/AgCl electrode as quasi-reference electrode (QRE). In all experiments, a 0.1 M tetrabutylammonium hexafluorophosphate ($TBAPF_6$, >99.0%, Sigma Aldrich) solution in dichloromethane (CH_2Cl_2, 99.7+%, Alfa Aesar) was used as electrolyte solution. The materials were dissolved therein exhibiting a resulting concentration of 1 mM. In addition, the electrolyte solution contained 0.1 mM ferrocene (98%, Sigma Aldrich) as internal standard.

The CV experiments were recorded in different runs; one time to the oxidative side and one time to the reductive side, where the actual potentials applied depended on the material. In all cases a scan rate of 200 mV s^{-1} was applied and the first cycle used for determination of the oxidation or reduction onset potential.

SEM records

Figure S5: SEM pictures of the flavin thin films surface with a magnification of 500× and 6000×.

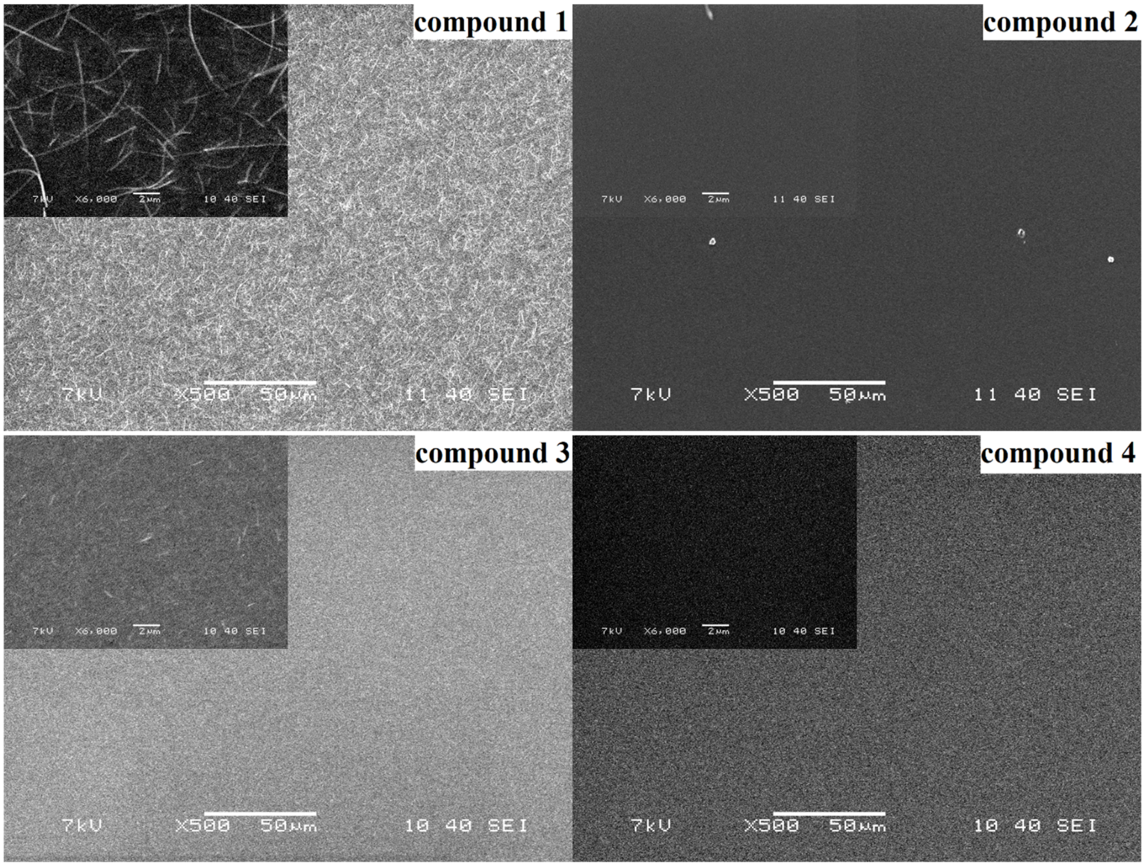

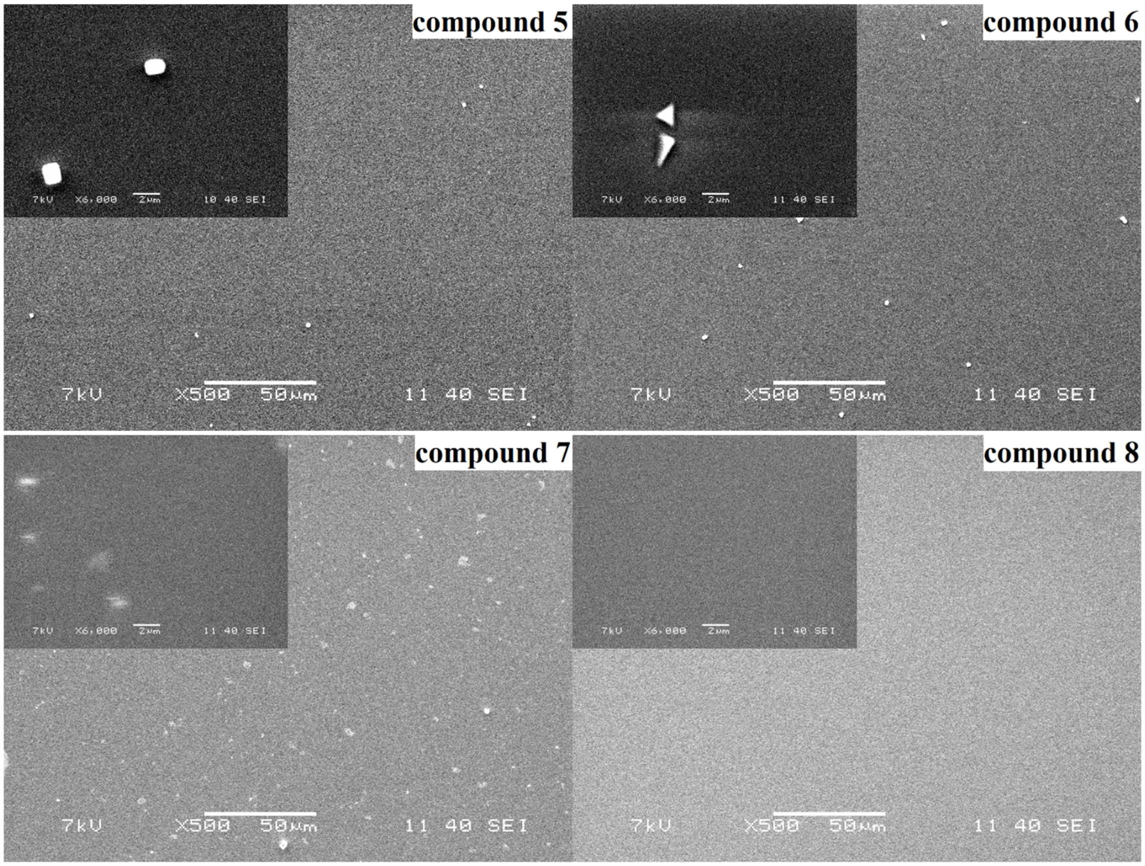

Flavins were vacuum deposited onto the ITO coated glass slides in the thickness of 150 nm utilizing PVD technique.

The scanning electron microscopy (SEM) images were obtained using JEOL JSM-6360 LV scanning electron microscope. High vacuum settings and an accelerating voltage of 7.0 kV were used.

Thin-film aging

Figure S6: The comparison of the absorption and emission spectra of flavin derivatives measured before and after the period of two months.

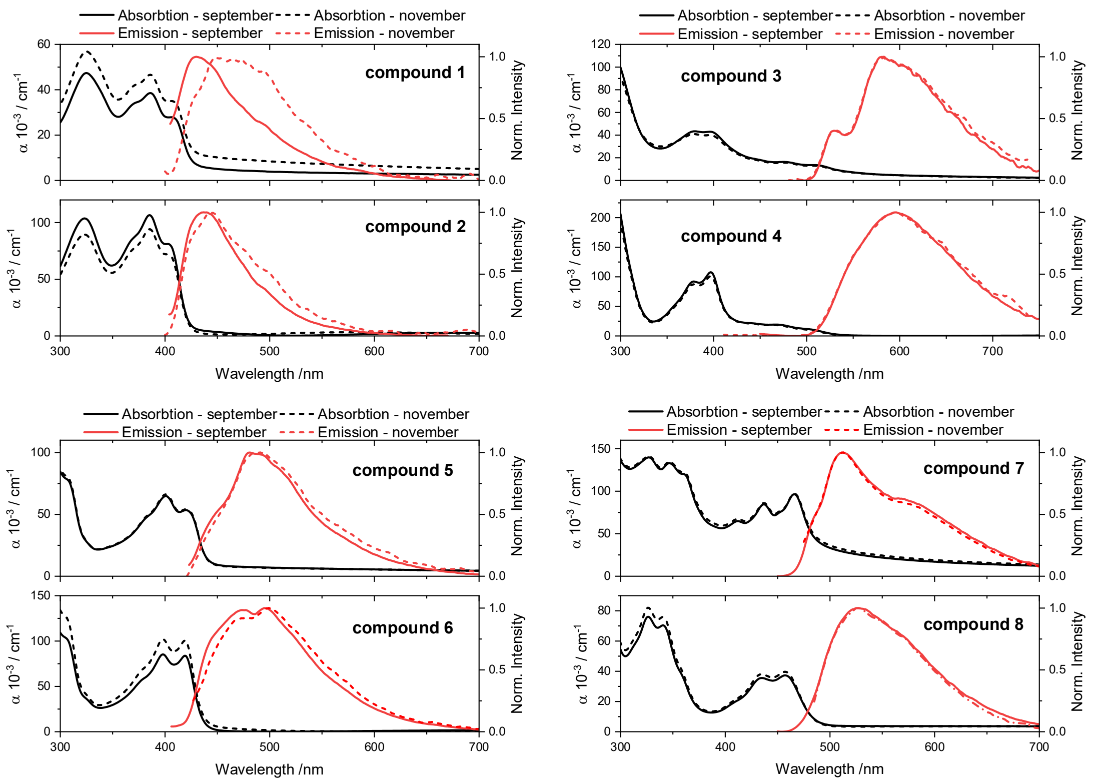

Figure S7: The comparison of the SEM scans of flavin derivatives measured before and after the period of two months with magnification of 2000×

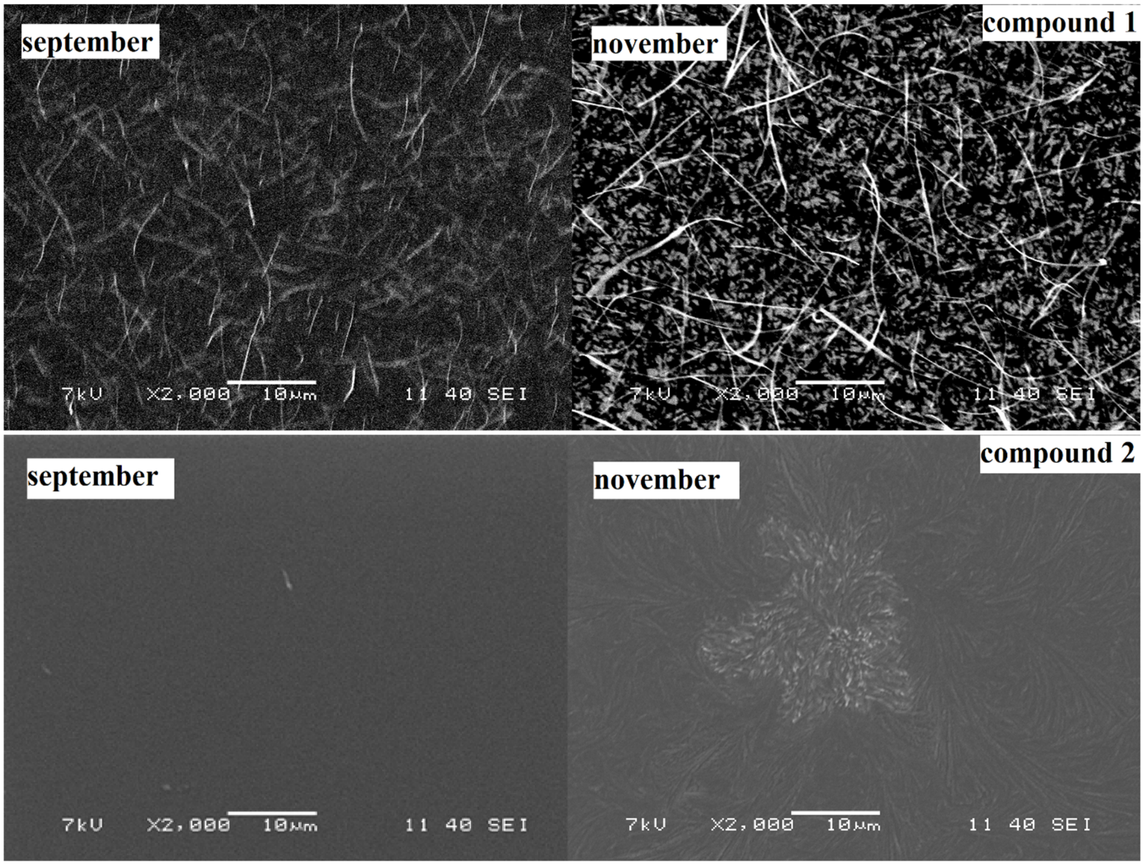

References

1. D. F. Swinehart. The Beer-Lambert Law. *J. Chem. Educ.* **1962**, *39*, 333.
2. H. Yang, T. J. Shin, M.-M. ling, K. Cho, C. Y. Ryu, Z. Bao. Conducting AFM and 2D GIXD Studies on Pentacene Thin Films. *J. Am. Chem. Soc.* **2005**, *127*, 11542.
3. C. Lee, W. Yang, R. G. Parr. Development of the Colle-Salvetti correlation-energy formula into a functional of the electron density. *Phys. Rev. B* **1988**, *37*, 785.
4. A. D. Becke. Density-functional thermochemistry. III. The role of exact exchange. *J. Chem. Phys.* **1993**, *98*, 5648.
5. V. Barone, M. Cossi. Quantum Calculation of Molecular Energies and Energy Gradients in Solution by a Conductor Solvent Model. *J. Phys. Chem. A* **1998**, *102*, 1995.
6. M. Cossi, N. Rega, G. Scalmani, V. Barone. Energies, structures, and electronic properties of molecules in solution with the C-PCM solvation model. *J. Comp. Chem.* **2003**, *24*, 669.
7. Gaussian 09, Revision C.01, M. J. Frisch, G. W. Trucks, H. B. Schlegel, G. E. Scuseria, M. A. Robb, J. R. Cheeseman, G. Scalmani, V. Barone, B. Mennucci, G. A. Petersson, H. Nakatsuji, M. Caricato, X. Li, H. P. Hratchian, A. F. Izmaylov, J. Bloino, G. Zheng, J. L. Sonnenberg, M. Hada, M. Ehara, K. Toyota, R. Fukuda, J. Hasegawa, M. Ishida, T. Nakajima, Y. Honda, O. Kitao, H. Nakai, T. Vreven, J. A. Montgomery, Jr., J. E. Peralta, F. Ogliaro, M. Bearpark, J. J. Heyd, E. Brothers, K. N. Kudin, V. N. Staroverov, T. Keith, R. Kobayashi, J. Normand, K. Raghavachari, A. Rendell, J. C. Burant, S. S. Iyengar, J. Tomasi, M. Cossi, N. Rega, J. M. Millam, M. Klene, J. E. Knox, J. B. Cross, V. Bakken, C. Adamo, J. Jaramillo, R. Gomperts, R. E. Stratmann, O. Yazyev, A. J. Austin, R. Cammi, C. Pomelli, J. W. Ochterski, R. L. Martin, K. Morokuma, V. G. Zakrzewski, G. A. Voth, P. Salvador, J. J. Dannenberg, S. Dapprich, A. D. Daniels, O. Farkas, J. B. Foresman, J. V. Ortiz, J. Cioslowski, and D. J. Fox, Gaussian, Inc., Wallingford CT, **2010**.
